# Surgical Management of Craniovertebral Junction Schwannomas: A Systematic Review

**DOI:** 10.3390/curroncol29070384

**Published:** 2022-07-09

**Authors:** Paolo Palmisciano, Gianluca Ferini, Gina Watanabe, Andie Conching, Christian Ogasawara, Gianluca Scalia, Othman Bin-Alamer, Ali S. Haider, Maurizio Passanisi, Rosario Maugeri, Samer S. Hoz, Matias Baldoncini, Alvaro Campero, Maurizio Salvati, Aaron A. Cohen-Gadol, Giuseppe E. Umana

**Affiliations:** 1 Department of Neurosurgery, University of Cincinnati College of Medicine, Cincinnati, OH 45267, USA; palmispo@ucmail.uc.edu; 2 Department of Radiation Oncology, REM Radioterapia srl, 95029 Viagrande, Italy; gianluca.ferini@grupposamed.com; 3 John A. Burns School of Medicine, University of Hawai’i, Honolulu, HI 96813, USA; ginaw@hawaii.edu (G.W.); andiec@hawaii.edu (A.C.); cogasawa@hawaii.edu (C.O.); 4 Department of Neurosurgery, Highly Specialized Hospital and of National Importance “Garibaldi”, 95122 Catania, Italy; gianluca.scalia@outlook.it; 5 Department of Neurosurgery, University of Pittsburgh Medical Center, Pittsburgh, PA 15213, USA; binalameroa@upmc.edu; 6 Department of Neurosurgery, The University of Texas M.D. Anderson Cancer Center, Houston, TX 77030, USA; aalam@mdanderson.org; 7 Trauma Center, Gamma Knife Center, Department of Neurosurgery, Cannizzaro Hospital, 95126 Catania, Italy; maurizio.passanisi@aoec.it; 8 Unit of Neurosurgery, Department of Experimental Biomedicine & Clinical Neuroscience, Azienda Ospedaliera Universitaria Policlinico, 90127 Palermo, Italy; rosario.maugeri1977@gmail.com; 9 Department of Neurological Surgery, Padilla Hospital, Tucumán T4000, Argentina; hozsr@ucmail.uc.edu (S.S.H.); alvarocampero@yahoo.com.ar (A.C.); 10 Department of Neurological Surgery, San Fernando Hospital, Buenos Aires B1646, Argentina; drbaldoncinimatias@gmail.com; 11 Department of Neurosurgery, Neuromed, IRCCS, Sapienza University of Rome, 86077 Pozzilli, Italy; maurizio.salvati@uniroma1.it; 12 Department of Neurological Surgery, Indiana University School of Medicine, Indianapolis, IN 46202, USA; acohenga@iu.edu

**Keywords:** cranial nerve tumor, craniovertebral junction, neuro-oncology, peripheral nerve tumor, schwannoma, skull base, spine

## Abstract

Background: Craniovertebral junction (CVJ) schwannomas are rare, with surgery and stereotactic radiosurgery (SRS) being effective yet challenging options. We systematically reviewed the literature on CVJ schwannomas. Methods: PubMed, Scopus, Web-of-Science, and Cochrane were searched following the PRISMA statement to include studies reporting CVJ schwannomas. Clinical features, management, and outcomes were analyzed. Results: We collected 353 patients from 101 included articles. Presenting symptoms were mostly neck pain (30.3%) and headache (26.3%), with most cranial neuropathies involving the XII (31.2%) and X (24.4%) nerves. Most tumors originated from C2 (30.9%) and XII (29.4%) nerves, being extracranial (45.1%) and intradural-extradural (44.2%). Erosion of C1–C2 vertebrae (37.1%), the hypoglossal canal (28.3%), and/or jugular foramen (20.1%) were noted. All tumors were operated, preferably with the retrosigmoid approach (36.5%), with the far-lateral approach (29.7%) or with the posterior approach and cervical laminectomy (26.9%), far-lateral approaches (14.2%), or suboccipital craniotomy with concurrent cervical laminectomy (14.2%). Complete tumor resection was obtained most frequently (61.5%). Adjuvant post-surgery stereotactic radiosurgery was delivered in 5.9% patients. Median follow-up was 27 months (range, 12–252). Symptom improvement was noted in 88.1% of cases, and cranial neuropathies showed improvement in 10.2%. Post-surgical complications occurred in 83 patients (23.5%), mostly dysphagia (7.4%), new cranial neuropathies (6.2%), and cerebrospinal fluid leak (5.9%). A total of 16 patients (4.5%) had tumor recurrence and 7 died (2%), with median overall survival of 2.7 months (range, 0.1–252). Conclusions: Microsurgical resection is safe and effective for CVJ schwannomas. Data on SRS efficacy and indications are still lacking, and its role deserves further evaluation.

## 1. Introduction

Schwannomas are heterogeneous tumors deriving from the myelinating Schwann cells of cranial and peripheral nerves. The 2021 WHO classification differentiates schwannomas from malignant nerve tumors, such as melanotic schwannomas and malignant peripheral nerve sheath tumors (MPNST), owing to their less aggressive nature and distinct genetic characteristics [1]. Schwannomas account for approximately 8% of nervous tumors, but their incidence depends on location and involved nerves, with facial and vestibular being the most common [2,3,4].

The craniovertebral junction (CVJ) defines the complex anatomical region incorporating the occiput and C1–C2 vertebrae [5,6]. Primary and metastatic neoplasms may infiltrate the CVJ challenging the surgical and non-operative treatment planning [7,8,9]. CVJ schwannomas are rare entities involving the jugular foramen, hypoglossal canal, or C1–C2 foramina, with direct extension to the atlanto-occipital and atlanto-axial joints [10]. Clinico-radiological presentations largely vary, with most tumors causing debilitating neuropathies and neurological impairments, and frequently presenting as contrast-enhancing dumbbell-shaped lesions [11,12]. Several surgical approaches have been described to optimize safe tumor dissection from the involved nerves, mostly by accurately exposing and controlling critical neurovascular structures [13,14]. Microsurgical resection represents the current standard to improve symptoms and local control, but stereotactic radiosurgery (SRS) may be considered in patients not eligible to undergo surgery and for treating post-surgical tumor remnants or recurrences [15,16]. Although effective, surgery and SRS correlate with some risks of severe complications.

The management of CVJ schwannomas constitutes a major topic of interest in skull base surgery, but available data are limited. In this study, we performed a systematic review of the literature to comprehensively describe the clinico-radiological characteristics, surgical management, and outcomes of patients with CVJ schwannomas.

## 2. Materials and Methods

### 2.1. Literature Search

A systematic review was performed upon the Preferred Reporting Items for Systematic Reviews and Meta-Analyses (PRISMA) statement [17,18] and registered to PROSPERO (ID: 331095). PubMed, Scopus, Web-of-Science, and Cochrane were searched from database inception to January 2022, operating the Boolean full-text search: ((schwannoma OR neurinoma OR neurilemmoma) AND (craniocervical OR craniovertebral junction OR jugular foramen OR hypoglossal OR C1 OR C2)). Collected studies were exported to Mendeley, and duplicates removed.

### 2.2. Study Selection

Pre-specified inclusion and exclusion criteria were set. Studies written in English were included if they involved ≥1 patients with histologically confirmed schwannomas extending into the CVJ region, as per the 2021 WHO classification [1]. CVJ schwannomas were defined as extramedullary lesions arising from the IX–X–XI cranial nerves on either the jugular foramen, the hypoglossal nerve near the hypoglossal canal, or C1–C2 nerve roots, and located infiltrating the CVJ region, as mentioned by the authors or deducted from imaging data. Studies were excluded if they: (1) were reviews, surgical videos, or autopsy reports; (2) reported patients with intramedullary cervical schwannomas, non-CVJ skull base schwannomas, or non-CVJ hypoglossal schwannomas (i.e., involving only the parapharyngeal, submandibular, or sublingual regions); (3) did not distinguish data of patients with CVJ schwannomas from data of patients with other tumors (e.g., meningiomas, melanocytic schwannomas, and MPNSTs).

Two authors (P.P. and C.O.) independently screened titles and abstracts of all studies and assessed the full texts of those meeting the inclusion criteria. Disagreements were solved by a third author (G.E.U.). Eligible articles were included. References were searched to retrieve additional relevant studies.

### 2.3. Data Extraction

Two authors (G.W. and A.C.) independently extracted data from included studies, which were then confirmed by one additional author (P.P.). Data were retrieved as explicitly reported by the authors of each included study, comprising: age, gender, nerve-of-origin, location, symptoms, cranial neuropathies, radiological findings, extent-of-resection, surgical approach, complications, adjuvant SRS, symptom improvement, recurrence, and survival status. Presenting symptoms referred to the patients’ clinical presentation observed at their admission. Cranial neuropathies defined the dysfunction of cranial nerves (i.e., in terms of motor and/or sensory function), as confirmed at the neurological examination performed pre-operatively during patients’ hospitalization. Extent-of-resection was defined as gross-total (GTR) for 100% tumor removal and partial (PTR) for less than 100% tumor removal.

### 2.4. Data Synthesis and Quality Assessment

Primary outcomes of interest were clinico-radiological presentation, management, and outcomes of CVJ schwannomas. For each study, two authors (P.P. and G.S.) independently evaluated level-of-evidence upon the 2011 Oxford Centre For Evidence-Based Medicine guidelines, and calculated risk of bias using the Joanna Briggs Institute checklists [19,20]. A study-level meta-analysis was precluded because all included articles had levels IV-V of evidence, and hazard ratios could not be deducted. Individual patient data were extracted for individual patient data meta-analysis.

## 3. Results

### 3.1. Study Selection

**Figure 1:**
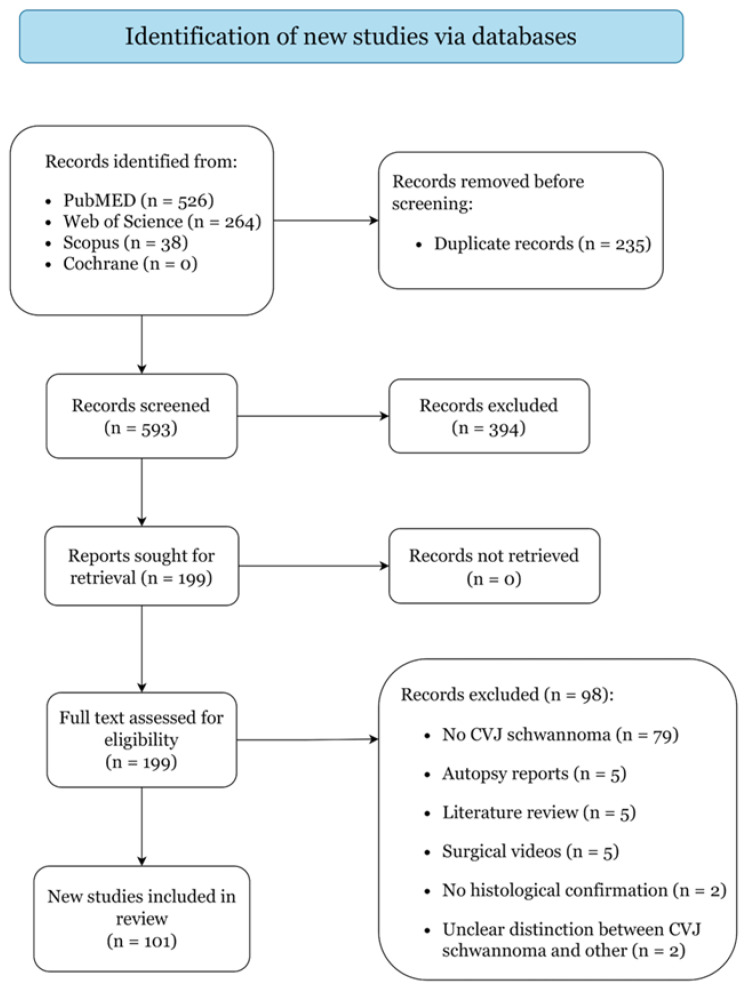
PRISMA 2020 Flow Diagram.

Figure 1 presents the study selection process. A total of 101 articles reporting on 353 cases were included: 32 case series and 69 case reports, categorized as level IV and V of evidence (Supplementary File S1). Quality assessment returned low risk of bias for all included studies (Supplementary File S2).

### 3.2. Patient Characteristics and Clinical Presentation

Median patient age was 49 years (range, 4–79) with no gender prevalence (Table 1). Neurofibromatosis was diagnosed in 11 patients: type-1 in 1 (0.3%) [21] and type-2 in 10 (2.8%) [10,12,22]. Median symptom duration was 6 months (range, 0–180). Clinical presentation mostly involved neck pain (30.3%), headache (26.3%), and sensory disturbances (26.1%). Motor disorders included limb weakness (10.8%), tetraparesis (7.9%), hemiparesis (5.7%), and paraparesis (4.8%). Cranial neuropathies mostly affected the XII (31.2%), X (24.4%), and IX (23.2%) nerves, and were multiple in 96 cases (27.2%). In 2 patients (0.6%) the CVJ masses were detected as incidental findings after neuroimaging exams performed for unrelated conditions [23,24].

### 3.3. Tumor Origin and Radiological Features

Most lesions were sited on the left (58.1%), and 6 patients (2%) had bilateral masses (Table 2). The nerve-of-origin was clearly identifiable at imaging in 295 cases (83.4%), with most lesions arising from C2 (30.9%) and XII (29.4%) nerves. For some lesions, exact tumor origin was not reported by the authors, with 44 tumors (12.5%) mentioned to originate from the IX–X–XI nerves, and 15 (4.2%) mentioned to originate from the C1–C2 nerves. Lesions were mostly extracranial (45.1%), with dumbbell-shaped intracranial–extracranial masses identified in 88 patients (27.6%). Additionally, most lesions were also intradural–extradural (44.2%). Most tumors infiltrated and/or eroded the C1–C2 vertebrae (37.1%), the hypoglossal canal (28.3%), and the jugular foramen (20.1%). Some masses caused occipital condyles erosion (18.7%), VA displacement (8.8%), brainstem compression (6.5%), 4th ventricle blockage (2.5%), and sigmoid sinus occlusion (1.1%). A total of 5 patients (1.4%) had concurrent CVJ intradural meningiomas separated from the extradural schwannomas [23,25,26,27], and 2 (0.6%) had acute subarachnoid hemorrhage (SAH) [28,29].

### 3.4. Management Strategies

All patients underwent microsurgical tumor resection, with GTR (61.5%) more frequent that PTR (38.5%) (Table 3). A total of 21 patients (5.9%) received adjuvant SRS for treating post-surgical remnant tumors. Most operations were performed with the retrosigmoid approach (36.5%) combined with suboccipital craniotomy (23.2%), with the far-lateral approach (29.7%) combined with suboccipital craniotomy and cervical laminectomy (15%), or with the posterior approach combined with cervical laminectomy (26.9%). The extreme lateral infrajugular transcondylar–transtubercular exposure (ELITE) was performed in 16 patients (4.5%) [30,31], and the endoscopic transoral resection in 8 (2.3%) [32,33,34]. Jiang et al. [11] also reported the use of occipitocervical fusion in 6 patients (1.7%) after cervical laminectomy and facetectomy. As regards intraoperative neuromonitoring adjuncts, monitoring on the lower cranial nerves (IX-XII) was used in 22 patients (6.2%) [35,36,37,38,39,40,41], monitoring of the somatosensory evoked potentials in 17 patients (4.8%) [11,21,42,43,44], monitoring of the motor evoked potential in 16 patients (4.5%) [11,21,42,45,46], and monitoring the brainstem evoked potential in 1 patient (0.3%) [38]. Similarly, microdoppler assistance was used only by Oichi et al. [26] to detect the relationship of the tumor and the surgical resection to the proximal arterial and venous structures in 1 patient (0.3%).

### 3.5. Treatment Outcomes

Median follow-up was 27 months (range, 12–252). Symptom improvement was reported in 88.1% cases. In addition, 37 patients (10.2%) with pre-operative cranial neuropathies experienced variable improvement in their cranial nerve function, with 7 (2%) experiencing complete resolution of their neuropathies and return to normal cranial nerve function (Table 4). Post-surgical complications occurred in 83 patients (23.5%), most commonly dysphagia (7.4%) and cerebrospinal fluid (CSF) leak (5.9%). New post-operative cranial neuropathies were found in 22 patients (6.2%), mostly involving the X (4%) and IX (2%) cranial nerves. Tumor recurrences occurred in 16 patients (4.5%) treated with surgery (56.3%), SRS (25%), or both (6.3%). A total of 7 patients (2%) died, 6 (1.7%) for unrelated medical and/or traumatic events, and 1 (0.3%) for post-operative dural sinus thrombosis and cerebellar infarction caused by over-coagulation of the mastoid emissary vein and venous plexus [47].

## 4. Discussion

The incidence of schwannomas is constantly increasing probably due to the widespread access to neurodiagnostic imaging and screening protocols in patients with neurological disorders, including headache and hearing disturbances [3]. Age at diagnosis is also rising, noted to be between the fourth and sixth decades of life in our pooled cohort and in patients with vestibular schwannomas [48]. Although the CVJ schwannomas are less frequent compared to vestibular and trigeminal schwannomas [3,49], such a rise in diagnoses has led to an increasing interest in better characterizing their clinical-radiological presentation and analyzing available management strategies.

The CVJ is a complex anatomical region of major interest in skull base and spine surgery. The occipital bone, including the foramen magnum, condyles, and lower clivus, the C1–C2 vertebrae, and the atlanto-occipital/atlanto-axial joints constitute the main structures, coupled with lower cranial and C1–C2 nerves, posterior vertebral circulation, and dural venous sinuses [5,6]. The adjacent brainstem and cervical spinal cord need also to be considered at clinical evaluation and surgical planning. In our pooled cohort, most tumors originated from the XII and C2 nerves, likely owing to their well-defined sensory nerve roots, which are deemed to be the site-of-origin of all schwannomas [12,22]. Yet, as reported in 16.6% of our cases, the origin of CVJ schwannomas may be difficult to ascertain at imaging and intraoperatively, due to the close proximity of contiguous nerve courses and the frequent tumor involvement of multiple bones [10]. Tumorigenesis remains unclear, with current theories focusing on “failure-of-nerve regeneration” mechanisms, characterized by injury-related *nf2* mutation and loss-of-function with upregulated proliferative signaling of Schwann cells [50]. CVJ schwannomas may originate from nerve injury caused by their exits through narrow skull base/vertebral bony foramina and adjacent pulsating arteries. The tumor microenvironment appears also to play a role, based on the presence of multiple cells within schwannomas, including macrophages and endothelial cells, that may modulate nerve degeneration and regeneration processes [51].

This between-cells interplay may be also accountable for the coexistence of concurrent meningiomas reported in patients with vestibular schwannomas and type-2 neurofibromatosis, and in 5 of our pooled patients [23,25,26,27,52,53]. The microenvironment of a preexisting neoplasm may accelerate the growth of the second by producing growth factors and mitogenic molecules [27]. However, other theories suggest a common mesenchymal progenitor cell between the two entities [26] and/or their occurrence as collision tumors, namely neighboring but separate lesions originating from different cells exposed to a single carcinogenic stimulus, such as local radiotherapy in Liebelt et al. [23]. Histological confirmation of both intradural (meningioma) and extradural (schwannoma) components is fundamental to differentiate these cases from the more common dumbbell-shaped CVJ schwannomas and devise appropriate treatments.

Clinical pictures of CVJ schwannomas mirror their anatomy, with most patients experiencing headache and neck pain as primary symptoms [10,12]. The high frequency of sensory disturbances likely stems from the sensory nerve roots’ tumor origin, while other impairments are caused by tumors compressing contiguous structures, such as lower cranial nerves (hoarseness, speech disorders, hearing disturbance), cerebellum (ataxia), and brainstem/cervical spinal cord (motor disorders) [31,36,54]. Tongue atrophy is typical in hypoglossal schwannomas [40], while evident neck masses may be found in otherwise asymptomatic slow-growing C1–C2 tumors [55]. Owing to the frequent multiple cranial neuropathies (27.1%) in our pooled cohort, we suggest performing comprehensive pre-operative neurological and radiological evaluations in patients with suspected CVJ masses to optimize the surgical planning and restore cranial nerves’ functions. At neuroimaging, CVJ schwannomas frequently caused enlargement and/or erosion of the C1–C2 foramen, hypoglossal canal, and jugular foramen, reflecting their most common nerves-of-origin. As in vestibular schwannomas, most lesions were dumbbell-shaped intradural–extradural and capsulated, while some masses extensively invaded the CVJ region and extended into the cervical spine canal, showing an intradural extramedullary location [28,44]. The close relationship between CVJ tumors and the arterio-venous structures requires pre-operative angiography or CT angiograms, which may largely aid the surgical planning [56,57]. In particular, these exams may show the VA course on the tumor’s surface and/or the tumor-related displacement/occlusion of the VA complex and venous sinuses, assisting the team to choose the optimal surgical routes for maximizing tumor exposure and vascular control [58].

The main therapeutic goals consist in providing clinical improvement and local tumor control, while minimizing complication risks and quality-of-life impairment [59,60,61]. Microsurgical tumor resection represents the current mainstay treatment, by allowing decompression of the CVJ neuro-vascular structures and prompt symptomatic relief. However, due to the complex surgical anatomy of these lesions, indications for tumor resection should be clearly defined at pre-treatment stages. Although the majority of our included articles failed to report tumor size and its impact on operative planning, we note that, across studies with available information, tumor resection was indicated in symptomatic patients with debilitating symptoms and/or neuropathies, or in asymptomatic patients with tumors compressing the brainstem and posing the risk to lead to life-threatening cardio-respiratory failure [10,62]. Future studies should better analyze the impact of tumor size on surgical planning, with the goal to better define the optimal indications to perform surgery for tumors originating from different cranial and cervical nerves.

As discussed in some of our pooled studies, we note that intraoperative neuromonitoring may represent a valuable assisting tool in CVJ schwannoma surgery, not just for nerve identification but also for real-time alert of neural compromise following excessive nerve traction or damage [21,39,42,63]. Similarly, micro-Doppler assistance may minimize the risk of vascular injuries in case of tumors encasing arterial and venous structures of the posterior circulation [10]. Although modern surgical management is shifting towards less aggressive procedures and PTR [59], most of our pooled cases received GTR, suggesting that accurate treatment planning and approach selection optimize safe tumor exposure and neuro-vascular control, with achievable maximal resection. No standardized guidelines on extent of surgical resection currently exist, but several authors suggested to pre-operatively plan PTR or GTR on a case-by-case basis [59]. As the surgical goal is to provide symptomatic relief and prevent the onset of tumor recurrence, while minimizing the risk of surgical complications, GTR should be planned only for tumors not encasing the vascular nor neural structures, especially if they have a clear surgical plane against the adjacent cranial nerves. Contrarily, in case of lesions invading or encasing critical neurovascular structures, where safe tumor removal is not pursuable, maximal PTR should be devised with the preservation of neurovascular structures infiltrated by the disease [59].

Surgical routes largely depended on tumor location and origin, with far-lateral, suboccipital, and retrosigmoid approaches preferred in the jugular foramen, foramen magnum, and hypoglossal canal tumors, while cervical laminectomy was frequently performed to better access C1–C2 tumors [10,12,31,36]. Tumors located in multiple compartments required combined skull base and spine approaches, such as the ELITE approach [30,31], to safely identify and dissect lesions from adjacent vascular and neural structures. The endoscopic transoral approach has also been reported by Crockard et al. [32] and Zhang et al. [33,34] as a viable route for the management of hypoglossal and cervical schwannomas. Although this approach allows maximal exposure of the anterior rim of the foramen magnum, of the occipital condyle, and of the C1 transverse process, the risks of spine instability and VA injury should be considered when drilling the anterior occipital condyles [33,34]. As reported by Jiang et al. [11] in their 6 cases, occipito-cervical fusion may be performed after CVJ schwannoma resection with C1–C2 facetectomy or occipital condylotomy to prevent spine instability, but higher risks of CSF leaks were noted. Intraoperatively, if nerve rootlets were embedded within tumors and not accurately detachable, nerves were often sacrificed to achieve GTR [64,65]. In these cases, patients experienced minimal or no neurological sequelae, likely because the presence of pre-operative deficits caused gradual compensation from adjacent neural structures and loss-of-function of the involved nerves [64].

Pooled post-surgery clinical outcomes proved that safe surgical resection leads to high rates of symptom improvement in patients with CVJ schwannomas, which may positively affect patients in their activities of daily living. As no information was currently available on health-related quality of life outcomes, future studies are advised to specifically focus on those outcomes, which could be collected by administering patient-oriented questionnaires at pre-operative and at post-operative stages, as currently done for other skull base surgeries [66]. Of interest, rates of post-surgery improvement in pre-operative cranial neuropathies were somewhat low (10.5%), likely due to the severe and irreversible nerve dysfunction cause by tumors’ within-nerve growth and disruption. The role of sural nerve grafting to the XII nerve has been described by Mathiesen et al. [67] (2 patients) and Nonaka et al. [30] (4 patients), showing good recovery (100% and 75%, respectively) of the XII nerve motor function at 1-year follow-up. Although the risks of some expected surgery-related complications may pose challenges during the pre-operative planning, we noted the occurrence of only modest rates of post-operative CSF leaks (5.9%) and new cranial neuropathies (6.2%) causing dysphagia (7.3%) and aspiration pneumonia (2.5%), which did not have a major impact on post-surgical outcomes. Finally, surgical resection, both PTR and GTR, allowed prolonged local tumor control and survival, with only 16 tumor recurrences/progression (4.5%) and 7 deaths (2%). While 6 deaths were not related to the pathology nor the operation, Hoshi et al. [47] reported 1 death in a patient with hypoglossal schwannoma caused by dural sinus occlusion and cerebellar infarct following the over-coagulation of the mastoid emissary vein and the venous plexus.

Although SRS has been accepted and regulated for vestibular schwannomas [3], only a few studies on CVJ schwannomas have been published, with limited information to devise standardized guidelines [59]. The largest-to-date multicenter studies from Hasegawa et al. [68] and Kano et al. [69] reported the use of upfront SRS for small non-dumbbell jugular foramen schwannomas and adjuvant STS for post-surgery residual tumors, showing variable rates of local tumor control, symptom relief, and cranial neuropathy improvement, but it somewhat risks radiation-induced adverse events. In our pooled cohort, 5.9% patients received adjuvant post-PTR SRS for the residual unresectable tumors; good clinical-radiological outcomes were noted, coupled with no post-radiotherapy complications [10,22]. Hence, SRS may play a major role in controlling small tumor remnants close to eloquent neuro-vascular structures, and thus should be considered at tumor resection stage to reduce the surgical invasiveness and plan post-operative sessions to maximize safe local tumor control. Future prospective studies should also analyze patient selection criteria for upfront SRS, especially for unresectable tumors.

### Limitations

Our review has some limitations. All included studies were retrospective case series exposed to selection bias and published within a 32-year time period characterized by major advances in surgical and radiotherapy protocols, which may have introduced some confounding variables into our analysis. The assessment of post-treatment clinical improvement and radiological response was subjective in most studies, and, owing to the limited patient-level data, we were not able to differentiate the types of post-surgical responses based on extent of tumor resection nor based on tumor type. Due to the lack of granular data found in the literature, we could not comprehensively assess differences in rates of local tumor control and survival between patients receiving adjuvant radiotherapy and patients receiving stand-alone surgery, nor analyze the impact of tumor size, histological grade, and subtypes on patients’ functional and survival outcomes. Finally, as we included only patients with a histologically confirmed diagnosis of primary SBCs, we could not compare the role of surgery vs. stand-alone radiotherapy.

## 5. Conclusions

CVJ schwannomas pose major challenges in planning best surgery and/or radiotherapy approaches. In this systematic review, we found that microsurgical resection is effective and safe after careful planning of optimal approaches. Some reports also suggested the use of intraoperative neuromonitoring and micro-Doppler assistance as valuable adjuncts in some complex cases. Post-surgical complications and neuropathies are worrisome, but sural nerve grafting may be useful for motor function recovery. The role of SRS for CVJ schwannomas deserves further evaluation, as it may offer an additional promising therapeutic option in patients not eligible to undergo surgery.

## Figures and Tables

**Table 1:** Summary of demographics and clinical presentation.

Characteristics	Value
Cohort size (no.)	353
Demographics	
Age (years), median (range)	46 (4–79)
Gender (male)	177 (50.1%)
Syndromes	No. (%)
Neurofibromatosis type 1	1 (0.3%)
Neurofibromatosis type 2	10 (2.8%)
Presenting Symptoms	No. (%)
Duration (months), median (range)	6 (0–180)
Neck pain	107 (30.3%)
Headache	93 (26.3%)
Sensory deficit	92 (26.1%)
Dysphagia/Swallowing difficulty	64 (18.1%)
Ataxia/Gait disturbance	62 (17.6%)
Hoarseness	52 (15.7%)
Motor weakness	38 (10.8%)
Speech disorder	38 (10.8%)
Tongue atrophy	35 (9.9%)
Hearing disturbance	35 (9.9%)
Vertigo	32 (9.1%)
Tetraparesis	28 (7.9%)
Diplopia	21 (5.9%)
Hemiparesis	20 (5.7%)
Paraparesis	17 (4.8%)
Neck mass	16 (4.5%)
No symptoms	2 (0.6%)
Cranial Nerve Neuropathies	No. (%)
V	7 (2%)
VII	24 (6.8%)
VIII	27 (7.6%)
IX	82 (23.2%)
X	86 (24.4%)
XI	30 (8.5%)
XII	110 (31.2%)
Multiple	96 (27.2%)

**Table 2:** Summary of tumor origin and radiological features.

Characteristics	Value
Tumor Laterality (*n* = 296)	No. (%)
Left	172 (58.1%)
Right	117 (39.5%)
Bilateral	6 (2%)
Midline	1 (0.3%)
Nerve of Origin	No. (%)
Cranial nerve IX	15 (4.2%)
Cranial nerve X	7 (2%)
Cranial nerve XI	15 (4.2%)
Cranial nerve IX–X–XI	44 (12.5%)
Cranial nerve XII	104 (29.4%)
Spinal nerve C1	46 (13%)
Spinal nerve C2	109 (30.9%)
Spinal nerve C1–C2	15 (4.2%)
Intracranial/Extracranial (*n* = 319)	No. (%)
Intracranial	87 (27.3%)
Extracranial	144 (45.1%)
Intracranial and Extracranial	88 (27.6%)
Intradural/Extradural (*n* = 319)	No. (%)
Intradural	129 (40.4%)
Extradural	49 (15.4%)
Intradural and Extradural	141 (44.2%)
Location	No. (%)
Jugular foramen	71 (86.5%)
Hypoglossal canal	100 (28.3%)
Jugular foramen and Hypoglossal canal	6 (1.7%)
Foramen magnum	5 (1.4%)
Foramen magnum and Hypoglossal canal	3 (0.8%)
Foramen magnum and C1	27 (7.6%)
Foramen magnum and C1–C2	7 (2%)
C1–C2 vertebra/foramen	131 (37.1%)
Radiological Findings	No. (%)
Erosion occipital condyle/cervical facet joint	66 (18.7%)
Displaced vertebral artery	31 (8.8%)
Compression brainstem	23 (6.5%)
Occlusion internal jugular vein/jugular bulb	20 (5.7%)
Invasion cerebellopontine angle/internal meatus	13 (3.7%)
Obstruction 4th ventricle	9 (2.5%)
Concurrent craniovertebral junction meningioma	5 (1.4%)
Occlusion sigmoid/transverse sinuses	4 (1.1%)
Subarachnoid hemorrhage	2 (0.6%)

**Table 3:** Summary of management strategies.

Characteristics	Value
Extent of Surgical Resection	No. (%)
Gross Total Resection (100%)	217 (61.5%)
Partial Resection (<100%)	136 (38.5%)
Surgical Approach (*n* = 334)	No. (%)
Retrosigmoid approach	129 (36.5%)
with suboccipital craniotomy	82 (23.2%)
with transcondylar craniotomy	26 (7.4%)
with suprajugular craniotomy	8 (2.3%)
with suboccipital and transcondylar craniotomy and cervical laminectomy	5 (1.4%)
with suboccipital and transcondylar craniotomy	4 (1.1%)
with cervical laminectomy	2 (0.6%)
with suboccipital craniotomy and cervical laminectomy	1 (7.6%)
with transcondylar and suprajugular craniotomy	1 (0.3%)
Far-lateral approach	105 (29.7%)
with suboccipital craniotomy and cervical laminectomy	53 (15%)
with suboccipital craniotomy	52 (14.7%)
Posterior approach with cervical laminectomy	95 (26.9%)
ELITE approach (Extreme lateral infrajugular transcondylar–transtubercular exposure)	16 (4.5%)
Transoral endoscopic approach	8 (2.3%)
Adjuvant Stereotactic Radiosurgery	21 (5.9%)

**Table 4:** Summary of treatment outcomes.

Characteristics	Value
Post-Surgical Complications	83 (23.5%)
Dysphagia	26 (7.4%)
Cerebrospinal fluid leak	21 (5.9%)
Aspiration pneumonia	9 (2.5%)
Hemiparesis/hemiplegia	9 (2.5%)
Hoarseness	8 (2.3%)
Hydrocephalus	4 (1.1%)
C2 anesthesia	3 (0.8%)
Wound infection	3 (0.8%)
Hearing loss	2 (0.6%)
Meningitis	2 (0.6%)
Subarachnoid hemorrhage	2 (0.6%)
Venous thrombosis	1 (0.3%)
Post-surgery New Cranial Nerve Neuropathies	No. (%)
Total	22 (6.2%)
VII	1 (0.3%)
IX	7 (2%)
X	14 (4%)
XI	6 (1.7%)
XII	7 (2%)
Symptom Improvement	311 (88.1%)
Improvement in pre-operative cranial nerve neuropathies	36 (10.2%)
Complete resolution	7 (2%)
Recurrence	16 (4.5%)
Status	No. (%)
Alive	346 (98%)
Dead	7 (2%)

## Supplementary Materials

The following supporting information can be downloaded at: https://www.mdpi.com/article/10.3390/curroncol29070384/s1, Supplementary File S1: Overview of all included studies, Supplementary File S2: Risk of bias assessments for all included studies.
