# Supplementary material for: Surgical Management of Craniovertebral Junction Schwannomas: A Systematic Review
Source: Curr Oncol. 2022 Jul 9;29(7):4842–55. doi: 10.3390/curroncol29070384 (PMC9319499; doi:10.3390/curroncol29070384)
Supplement: Supplementary file 1 [file curroncol-29-00384-s001.zip › Supplementary File S1.pdf]

Supplementary File S1. Overview of all included studies.

Authors – Year	Study Design – Level of Evidence	Patients No./ Male	Age years median (range)	Cranial Nerve Deficits at Presentation No. patients (percentage)	Tumor Origin No. patients (percentage)	Laterality No. patients (percentage)	Location No. patients (percentage)	Tumor Type No. patients (percentage)	Surgical Approach No. patients (percentage)	Extent of Resection No. patients (percentage)	Complications No. patients (percentage)	Stereotactic Radiosurgery No. patients (percentage)	Symptom Improvement No. patients (percentage)	Post-Operative Cranial Nerve Deficits No. patients (percentage)	Recurrence No. patients (percentage)	PFS months median (range)	OS months median (range)	Status No. patients (percentage)
**Scott et al. – 1949[1]**	Case report – V	1 / 0 (0%)	50	IX, X, XI, XII	IX-X-XI	R	JF & HC	I, ID	SC + CL (C1-C3	PTR	0 (0%)	0 (0%)	1 (100%)	IX, X, XI, XII	0 (0%)		36.0	1 Alive (100%)
**Morelli et al. – 1966[2]**	Case report – V	1 / 0 (0%)	22	XII	XII	R	HC	I, ID	SC + CL (C1)	GTR	0 (0%)	0 (0%)	0 (0%)	XII	0 (0%)		0.1	1 Dead (100%)
**Strang et al. – 1967[3]**	Case report – V	1 / 0 (0%)	51	IX, X, XI, XII	XII	R	HC	I-E, ID-ED	SC + CL (C1)	GTR	0 (0%)	0 (0%)	1 (100%)	XII	0 (0%)		3.0	1 Alive (100%)
**Bartal et al. – 1973[4]**	Case report – V	1 / 1 (100%)	36	X, XI, XII	XII	L	HC	I-E, ID-ED	SC	PTR	Hydrocephalus	0 (0%)	1 (100%)	XII	0 (0%)		12.0	1 Alive (100%)
**Sumra et al. – 1978[5]**	Case report – V	1 / 1 (100%)	46	0 (0%)	C2	R	C1-C2 F	E, ID-ED	CL (C2)	GTR	0 (0%)	0 (0%)	1 (100%)	0 (0%)	0 (0%)		1.0	1 Alive (100%)
**Conrad et al. – 1979[6]**	Case report – V	1 / 0 (0%)	46	0 (0%)	C2	L	C1-C2 F	E, ID	CL (C2)	GTR	0 (0%)	0 (0%)	1 (100%)	0 (0%)	0 (0%)		1.0	1 Alive (100%)
**Fujiwara et al. – 1980[7]**	Case report – V	1 / 0 (0%)	45	XII	XII	R	HC	I, ID	SC	GTR	0 (0%)	0 (0%)	1 (100%)	XII	0 (0%)		3.0	1 Alive (100%)
**Ulsø et al. – 1981[8]**	Case report – V	1 / 0 (0%)	28	V, VII, IX, X, XI, XII	XII	L	FM	I, ID	SC	GTR	0 (0%)	0 (0%)	1 (100%)	XII	0 (0%)		4.0	1 Alive (100%)
**Berger et al. – 1982[9]**	Case report – V	1 / 0 (0%)	17	XII	XII	R	HC	I, ID	SC	GTR	0 (0%)	0 (0%)	1 (100%)	XII	0 (0%)		3.0	1 Alive (100%)
**Dolan et al. – 1982[10]**	Case report – V	1 / 0 (0%)	24	VII, IX, X, XI, XII	XII	L	HC	I-E, ID	SC	PTR	CSF leak	0 (0%)	1 (100%)	IX, X, XII	0 (0%)		3.0	1 Alive (100%)
**Hakuba et al. – 1984[11]**	Case series – IV	2 / 1 (50%)	55	0 (0%)	2 C2 (100%)	1 L (50%) 1 R (50%)	2 C1-C3 F (100%)	2 I-E, ID-ED (100%)	2 CL (C2)	2 PTR (100%)	0 (0%)	0 (0%)	2 (100%)	0 (0%)	0 (0%)		21.0 (12-30)	2 Alive (100%)
**Tuck et al. – 1984[12]**	Case series – IV	2 / 0 (0%)	57 (52-62)	1 XI, XII (50%) 1 VIII, IX, X, XII (50%)	2 XII (100%)	1 L (50%) 1 R (50%)	2 HC (100%)	1 I, ID (50%) 1 I-E, ID-ED (50%)	2 SC (100%)	1 PTR (50%) 1 GTR (50%)	0 (0%)	0 (0%)	2 (100%)	1 XI, XII (50%) 1 XII (50%)	1 (50%)	36.0	30.0 (12-48)	2 Alive (100%)
**Bettoni et al. – 1985[13]**	Case report – V	1 / 0 (0%)	62	VII, XII	XII	L	HC	I, ED	SC	GTR	0 (0%)	0 (0%)	1 (100%)	XII	0 (0%)		24.0	1 Alive (100%)
**Lesoin et al. – 1986[14]**	Case report – V	1 / 1 (100%)	55	0 (0%)	C2	R	C1-C3 F	E, ED	CL (C2-C3)	PTR	C2 anesthesia	0 (0%)	1 (100%)	0 (0%)	0 (0%)		3.0	1 Alive (100%)
**Shearman et al. – 1986[15]**	Case report – V	1 / 1 (100%)	4	XI, XII	XII	L	JF	I-E, ED	SC	GTR	0 (0%)	0 (0%)	1 (100%)	XI, XII	1 (100%)	8.0	12.0	1 Alive (100%)
**Solymosi et al. – 1987[16]**	Case report – V	1 / 1 (100%)	48	VIII, IX, X, XII	XII	L	JF	I, ID-ED	SC	GTR	0 (0%)	0 (0%)	1 (100%)	XII	0 (0%)		3.0	1 Alive (100%)
**Nakashima et al. – 1988[17]**	Case report – V	1 / 1 (100%)	67	VIII, IX, X, XI, XII	XI	R	JF	I, ID-ED	SC-RS	PTR	0 (0%)	0 (0%)	1 (100%)	IX, X	0 (0%)		10.0	1 Alive (100%)
**Crockard et al. – 1991[18]**	Case series – IV	2 / 2 (100%)	39 (32-46)	0 (0%)	2 C1 (100%)	2 R (100%)	2 FM (100%)	2 I-E, ID (100%)	2 Transoral (100%)	2 GTR (100%)	2 Dysphagia (100%)	0 (0%)	1 (100%)	0 (0%)	0 (0%)		54.0 (36-72)	2 Alive (100%)
**Kratimenos et al. – 1993[19]**	Case series – IV	4 / 1 (25%)	30.5 (24-34)	0 (0%)	4 C1 (100%)	3 L (75%) 1 R (25%)	2 C1-C2 F (50%) 1 FM (25%)	4 E, ID-ED (100%)	4 FL (100%)	4 GTR (100%)	0 (0%)	0 (0%)	4 (100%)	0 (0%)	0 (0%)		3.0	4 Alive (100%)

							1 FM-C1 (25%)											
**Krauss et al. – 1993[20]**	Case report – V	1 / 0 (0%)	49	V, VII, IX, X, XII	XII	R	HC	I, ID	SC-TC	PTR	Dysphagia, pneumonia	0 (0%)	1 (100%)	IX, X	1 (100%)	2.0	3.0	1 Dead (100%)
**Kamitani et al. – 1994[21]**	Case series – IV	2 / 1 (50%)	50.5 (48-53)	1 VII, VIII (50%) 1 XII (50%)	1 IX-X-XI (50%) 1 XII (50%)	2 L (100%)	1 JF (50%) 1 JF & HC (50%)	2 I-E, ID-ED (100%)	1 SC (50%) 1 SC + CL (C1-C2) (50%)	1 PTR (50%) 1 GTR (50%)	1 Hemiplegia (50%)	0 (0%)	1 (50%)	1 VII, VIII (50%) 1 IX, X, XI, XII (50%)	1 (50%)	24.0	14.5 (3-26)	2 Alive (100%)
**Okura et al. – 1994[22]**	Case report – V	1 / 0 (0%)	59	XII	XII	R	HC	I-E, ID-ED	SC	GTR	0 (0%)	0 (0%)	1 (100%)	XI, XII	0 (0%)		2.0	6 Alive (30%)
**Smith et al. – 1995[23]**	Case report – V	1 / 1 (100%)	47	IX, X, XII	XII	L	HC	I-E, ID-ED	TC	GTR	0 (0%)	0 (0%)	1 (100%)	IX, X, XII	0 (0%)		3.0	1 Alive (100%)
**Kobayashi et al. – 1996[24]**	Case report – V	1 / 0 (0%)	46	0 (0%)	XII	R	HC	I, ID	SC	GTR	CSF leak	0 (0%)	1 (100%)	IX, X, XI, XII	0 (0%)		3.0	1 Alive (100%)
**Sato et al. – 1996[25]**	Case report – V	1 / 0 (0%)	59	XII	XII	R	HC	I-E, ID-ED	SC-TC + CL (C1)	PTR	CSF leak	0 (0%)	1 (100%)	XII	0 (0%)		1.0	1 Alive (100%)
**Sairyo et al. – 1997[26]**	Case report – V	1 / 1 (100%)	64	0 (0%)	C2	R	C1-C2 F	E, ED	CL (C1)	GTR	0 (0%)	0 (0%)	1 (100%)	0 (0%)	0 (0%)		3.0	1 Alive (100%)
**Bunc et al. – 1998[27]**	Case report – V	1 / 1 (100%)	53	IX, X, XII	XII	R	HC	I-E, ID-ED	SC-TC	GTR	Dysphagia	0 (0%)	1 (100%)	IX, X, XI, XII	0 (0%)		12.0	1 Alive (100%)
**Gilsbach et al. – 1998[28]**	Case report – V	1 / 1 (100%)	48	IX, XII	XII	R	JF & HC	I, ID	SC	GTR	0 (0%)	0 (0%)	1 (100%)	IX, XI, XII	0 (0%)		12.0	1 Alive (100%)
**Spinnato et al. – 1998[29]**	Case report – V	1 / 0 (0%)	49	0 (0%)	XII	R	FM & HC	I-E, ID-ED	SC-TC + CL (C1-C2)	GTR	0 (0%)	0 (0%)	1 (100%)	0 (0%)	0 (0%)		3.0	1 Alive (100%)
**Kachhara et al. – 1999[30]**	Case report – V	1 / 1 (100%)	24	XII	XII	R	HC	I-E, ID-ED	SC	PTR	0 (0%)	0 (0%)	1 (100%)	XII	0 (0%)		3.0	1 Alive (100%)
**Hoshi et al. – 2000[31]**	Case series – IV	2 / 2 (100%)	55.5 (48-63)	1 IX, XII (50%) 1 VII, IX, X, XI, XII (50%)	2 XII (100%)	2 R (100%)	2 HC (100%)	2 I-E, ID-ED (100%)	2 SC (100%)	1 PTR (50%) 1 GTR (50%)	1 SAH (50%)	0 (0%)	2 (100%)	1 XII (50%) 1 IX, X (50%)	0 (0%)		1 (0.1-2)	1 Alive, 1 Dead (50%)
**Kalavakonda et al. – 2000[32]**	Case report – V	1 / 1 (100%)	52	0 (0%)	C1	L	FM-C1	E, ED	TC	GTR	0 (0%)	0 (0%)	1 (100%)	0 (0%)	0 (0%)		3.0	1 Alive (100%)
**Mariniello et al. – 2000[33]**	Case report – V	1 / 0 (0%)	20	0 (0%)	XII	R	HC	I-E, ED	SC	GTR	0 (0%)	0 (0%)	1 (100%)	IX, X, XI, XII	0 (0%)		24.0	1 Alive (100%)
**Kyoshima et al. – 2003[34]**	Case series – IV	2 / 0 (0%)	65 (57-73)	0 (0%)	2 C2 (100%)	1 L (50%) 1 R (50%)	2 C1-C2 F (100%)	2 E, ID-ED (100%)	2 CL (C1-C2) (100%)	2 GTR(100%)	0 (0%)	0 (0%)	2 (100%)	0 (0%)	0 (0%)		24.0	2 Alive (100%)
**Leal Filho et al. – 2003[35]**	Case series – IV	2 / 1 (50%)	51 (43-59)	1 IX, X (50%)	C1	2 R (100%)	2 FM-C1 (100%)	2 E, ID-ED (100%)	2 FL (100%)	2 GTR (100%)	0 (0%)	0 (0%)	2 (100%)	0 (0%)	0 (0%)		6.0	2 Alive (100%)
**Passacantilli et al. – 2003[36]**	Case report – V	1 / 1 (100%)	44	0 (0%)	XII	L	HC	I-ED	FL	PTR	0 (0%)	0 (0%)	1 (100%)	0 (0%)	0 (0%)		3.0	1 Alive (100%)
**Tatebayashi et al. – 2003[37]**	Case report – V	1 / 0 (0%)	46	XI	XI	R	JF	I, ID	SC + CL (C1)	GTR	0 (0%)	0 (0%)	1 (100%)	XI	0 (0%)		24.0	1 Alive (100%)
**Aihara et al. – 2004[38]**	Case report – V	1 / 0 (0%)	72	XII	XII	R	HC	I-E, ID-ED	FL	PTR	0 (0%)	0 (0%)	1 (100%)	XII	0 (0%)		3.0	1 Alive (100%)
**Ho et al. – 2004[39]**	Case report – V	1 / 1 (100%)	39	XII	XII	R	FM & HC	I-E, ID-ED	SC-TC	GTR	0 (0%)	0 (0%)	1 (100%)	XII	0 (0%)		12.0	1 Alive (100%)
**Kadri et al. – 2004[40]**	Case series – IV	6 / 3 (50%)	31 (23-42)	6 IX, X (100%) 4 XI, XII (66.7%) 2 VIII (33.3%) 1 V, VI, VII (16.7%)	3 X (50%) 3 IX-X-XI (50%)	4 L (66.7%) 2 R (33.3%)	6 JF (100%)	6 I-E, ID-ED (100%)	6 TC + SJ (83.3%) 1 TC (16.7%)	6 GTR (100%)	0 (0%)	0 (0%)	6 (100%)	6 IX, X (100%) 4 XI, XII (66.7%) 2 VIII (33.3%) 1 V, VI (16.7%)	0 (0%)		30.0 (2-66)	6 Alive (100%)
**Kim et al. – 2005[41]**	Case report – V	1 / 0 (0%)	60	0 (0%)	C1	R	FM-C1	E	FL + CL (C1)	PTR	0 (0%)	1 (100%)	1 (100%)	0 (0%)	0 (0%)		2.0	1 Alive (100%)
**Takumi et al. – 2005[42]**	Case report – V	1 / 1 (100%)	35	0 (0%)	C1	R	FM-C1	E, ID	SC-RS	GTR	0 (0%)	0 (0%)	1 (100%)	0 (0%)	0 (0%)		48.0	1 Alive (100%)
**Sato et al. – 2006[43]**	Case report – V	1 / 0 (0%)	54	XII	XII	L	HC	I-E, ID-ED	SC	PTR	0 (0%)	0 (0%)	1 (100%)	0 (0%)	0 (0%)		24.0	1 Alive (100%)
**Ueda et al. – 2006[44]**	Case series – IV	3 / 0 (0%)	54 (50-79)	0 (0%)	3 C1 (100%)	3 R (100%)	3 FM-C1 (100%)	3 E, ID (100%)	3 SC-TC + CL (C1-C2)	3 GTR (100%)	1 CSF leak (33.3%)	0 (0%)	1 (100%)	0 (0%)	0 (0%)		79 (38-88)	3 Alive (100%)
**Kikkawa et al. – 2007[45]**	Case report – V	1 / 0 (0%)	36	VII, IX, X	XII	L	FM-C1	I, ID	SC + CL (C1)	GTR	0 (0%)	0 (0%)	1 (100%)	XII	0 (0%)		3.0	1 Alive (100%)
**Kim et al. – 2007[46]**	Case report – V	1 / 0 (0%)	68	XII	XII	L	HC	I, ID	SC-RS	GTR	0 (0%)	0 (0%)	1 (100%)	XII	0 (0%)		6.0	1 Alive (100%)
**Tucker et al. – 2007[47]**	Case report – V	1 / 0 (0%)	31	XII	XII	L	HC	I-E, ID-ED	SC	PTR	Dysphagia	0 (0%)	1 (100%)	XII	1 (100%)	15.0	27.0	1 Alive (100%)
**Ciappetta et al. – 2008[48]**	Case report – V	1 / 0 (0%)	44	XI	C2	L	C1-C2 F	I-E, ID	CL (C1-C5)	GTR	0 (0%)	0 (0%)	1 (100%)	0 (0%)	0 (0%)		6.0	1 Alive (100%)
**Garg et al. – 2008[49]**	Case series – IV	1 /	35	XII	XII	R	HC	I-E, ID-ED	SC-RS	PTR	Hemiplegia	0 (0%)	1 (100%)	XII	1 (100%)	1.0	2.0	1 Dead

		0 (0%)																(100%)
**Kuo et al. – 2008[50]**	Case report – V	1 /<br>0 (0%)	25	XII	XII	R	HC	I-E, ED	FL-SC	PTR	0 (0%)	0 (0%)	0 (0%)	XII	0 (0%)		6.0	1 Alive<br>(100%)
**Chibbaro et al. – 2009[51]**	Case series – IV	16 /<br>3 (18.8%)	41<br>(16-71)	5 IX, X (31.3%)<br>3 IX, X, XI (18.2%)<br>2 X (12.5%)<br>1 V (6.3%)<br>1 V, X (6.3%)<br>1 VIII, IX, X, XI<br>(6.3%)	7 IX-X-XI<br>(43.7%)<br>7 IX (43.7%)<br>1 X (6.3%)<br>1 XI (6.3%)	9 R<br>(56.3%)<br>7 L<br>(43.7%)	16 JF<br>(100%)	13 I (81.3%)<br>3 E (18.7%)	16 TC<br>(100%)	13 GTR<br>(81.3%)<br>3 PTR<br>(18.7%)	4 Dysphagia<br>(25%)<br>1 CSF leak<br>(6.3%)<br>1 Aspiration<br>pneumo<br>(6.3%)	0 (0%)	16 (100%)	5 IX, X (31.3%)<br>2 IX, X, XI<br>(12.5%)	0 (0%)		94<br>(24-252)	16 Alive<br>(100%)
**Heiroth et al. – 2009[52]**	Case report – V	1 /<br>0 (0%)	23	XII	XII	R	HC	E, ED	FL	GTR	Hoarseness	0 (0%)	1 (100%)	X, XII	0 (0%)			1 Alive<br>(100%)
**Ichimura et al. – 2009[53]**	Case series – IV	7 /<br>3 (42.9%)	50<br>(38-71)	3 IX, X, XII<br>(42.9%)<br>2 XII (28.6%)<br>2 IX, X, XI, XII<br>(28.6%)	7 XII (100%)	N/A	7 HC<br>(100%)	6 I-E, ID-ED<br>(85.7%)<br>1 E, ED<br>(14.3%)	7 SC + CL<br>(C1) (100%)	4 PTR<br>(66.7%)<br>2 GTR<br>(33.3%)	1 Venous<br>thrombosis<br>(14.3%)	3 (42.9%)	7 (100%)	7 XII (100%)	0 (0%)		6.0	6 Alive<br>(85.7%)<br>1 Dead<br>(14.3%)
**Mathiesen et al. – 2009[54]**	Case series – IV	2 /<br>1 (50%)	53	2 XII (100%)	2 XII (100%)	2 L (100%)	2 HC<br>(100%)	2 I-E, ID-ED<br>(100%)	2 RS + CL<br>(C1) (100%)	2 GTR<br>(100%)	1 CSF leak,<br>dysphagi<br>(50%)	0 (0%)	2 (100%)	1 IX, X, XII<br>(50%)	0 (0%)		15.0<br>(6-24)	2 Alive<br>(100%)
**Watanabe et al. – 2009[55]**	Case series – IV	9 /<br>6 (66.7%)	54<br>(22-68)	0 (0%)	5 C2 (55.6%)<br>4 C1 (44.4%)	N/A	9 C1-C2 F<br>(55.6%)	9 E, ID-ED<br>(100%)	9 SC (100%)	9 PTR<br>(100%)	1 CSF leak<br>(11.1%)	0 (0%)	9 (100%)	0 (0%)	2 (22.2%)	12.0	30.0<br>(7-180)	9 Alive<br>(100%)
**Edizer et al. – 2010[56]**	Case report – V	1 /<br>0 (0%)	34	0 (0%)	XII	R	FM	E, ED	CL (C1)	GTR	0 (0%)	0 (0%)	0 (0%)	XII	0 (0%)		3.0	1 Alive<br>(100%)
**Ishikawa et al. – 2010[57]**	Case report – V	1 /<br>1 (100%)	54	X, XII	XII	L	HC	I, ID	SC-TC	PTR	0 (0%)	0 (0%)	0 (0%)	X, XII	0 (0%)		12.0	1 Alive<br>(100%)
**Ji et al. – 2010[58]**	Case report – V	1 /<br>1 (100%)	44	0 (0%)	C1-C2	L	C1-C2 F	E, ID	CL (C1-C2)	GTR	0 (0%)	0 (0%)	1 (100%)	0 (0%)	0 (0%)		3.0	1 Alive<br>(100%)
**Li et al. – 2010[59]**	Case report – V	1 /<br>0 (0%)	36	0 (0%)	XII	R	FM & HC	I, ID	SC	PTR	0 (0%)	0 (0%)	1 (100%)	XII	1 (100%)	20.0	24.0	1 Alive<br>(100%)
**Cavalcanti et al. – 2010[60]**	Case series – IV	36 /<br>19<br>(52.8%)	43<br>(17-68)	5 X (13.9%)<br>1 XII (2.8%)<br>1 VII, VIII (2.8%)<br>1 XI, XII (2.8%)<br>1 V, IX, X (2.8%)<br>1 VII,VIII,X (2.8%)<br>1 IX, XI, XI (2.8%)<br>1 VII, VIII, IX, X<br>(2.8%)<br>1 VIII, IX, X, XII<br>(2.8%)<br>1 IX, X, XI, XII<br>(2.8%)	17 IX-X-XI<br>(47.2%)<br>13 C1-C2<br>(36.1%)<br>6 XII (16.7%)	14 L<br>(38.9%)<br>9 R (25%)	17 JF<br>(47.2%)<br>13 C1-C2 F<br>(36.1%)<br>6 HC<br>(16.7%)	16 ED-ID<br>(44.4%)<br>15 ID<br>(41.7%)<br>5 ED<br>(13.9%)<br>10 I (10<br>(27.8%)<br>4 I-E<br>(11.1%)<br>2 E (5.6%)	20 FL<br>(55.6%)<br>13 CL (C1-<br>C2) (36.1%)<br>8 RS<br>(22.2%)	25 GTR<br>(69.4%)<br>11 PTR<br>(30.6%)	8 Dysphagia<br>(22.2%)<br>3 Aspiration<br>pneumonia<br>(8.3%)<br>3 CSF leak<br>(8.3%)<br>1Hydrocepha-<br>lus (2.8%)<br>1 Meningitis<br>(2.8%)	8 (22.2%)	19 (52.8%)	7 X (19.4%)<br>3 XII (8.3%)<br>3 IX, X, XI<br>(8.3%)<br>1 IX (2.8%)<br>1 VII,VIII<br>(2.8%)<br>1 VII, IX, X<br>(2.8%)	1 (2.8%)	12	25<br>(0-110)	35 Alive<br>(97.2%)<br>1 Dead<br>(2.8%)
**Takami et al. – 2010[61]**	Case series – IV	8 /<br>1 (12.5%)	49.5<br>(19-65)	0 (0%)	7 C2 (87.5%)<br>1 C1 (12.5%)	N/A	8 C1-C2 F<br>(100%)	8 E, ID-ED<br>(100%)	8 CL (C1-<br>C2) (100%)	8 GTR<br>(100%)	0 (0%)	0 (0%)	8 (100%)	0 (0%)	0 (0%)		3.0	8 Alive<br>(100%)
**Gopalakrishnam et al. – 2011[62]**	Case report – V	1 /<br>1 (100%)	40	0 (0%)	C1	M	FM-C1<br>(100%)	I-E, ID-ED	SC	GTR	0 (0%)	0 (0%)	1 (100%)	0 (0%)	0 (0%)		1.0	1 Alive<br>(100%)
**Ramzan et al. – 2011[63]**	Case report – V	1 /<br>0 (0%)	14	0 (0%)	C1-C2	L	FM-C2	I-E, ID-ED	SC + CL<br>(C1-C3)	PTR	0 (0%)	0 (0%)	1 (100%)	0 (0%)	0 (0%)		3.0	1 Alive<br>(100%)
**Nonaka et al. – 2011[64]**	Case series – IV	13 /<br>8 (61.5%)	44<br>(13-71)	6 XII (46.1%)<br>3 IX, X, XII<br>(23.1%)<br>1 IX, X (7.7%)	13 XII (100%)	11 R<br>(84.6%)<br>2 L<br>(15.4%)	13 HC<br>(100%)	7 I-E, ID-<br>ED (53.8%)<br>5 I, ID<br>(38.5%)<br>1 E, ED<br>(7.7%)	13 ELITE<br>(100%)	10 GTR<br>(76.9%)<br>3 PTR<br>(23.1%)	4 Hoarseness<br>(30.8%)<br>3 Dysphagia<br>(23.1%)<br>1 CSF leak<br>(7.7%)	0 (0%)	13 (100%)	6 XII (46.1%)<br>4 X (30.8%)<br>3 IX, X (23.1%)<br>3 IX, X, XII<br>(23.1%)<br>1 VII (7.7%)	1 (7.7%)	48	6<br>(9-168)	13 Alive<br>(100%)
**Wang et al. – 2011[65]**	Case series – IV	18 /<br>10<br>(55.6%)	47<br>(24-72)	0 (0%)	16 C2 (88.9%)<br>2 C1 (11.1%)	N/A	18 C1-C2 F<br>(100%)	14 E, ID-ED<br>(77.8%)<br>4 E, ED<br>(22.2%)	18 FL<br>(100%)	15 GTR<br>(83.3%)<br>3 PTR<br>(16.7%)	2 CSF leak<br>(11.1%)<br>1 Infection<br>wound (5.5%)	0 (0%)	12 (66.7%)	0 (0%)	1 (5.6%)	48	43<br>(3-110)	17 Alive<br>(94.4%)<br>1 Dead<br>(5.6%)
**Zhang et al. – 2011[66]**	Case series – IV	3 /<br>1 (33.3%)	59<br>(58.61)	2 XII (66.7%)<br>1 IX, X, XII<br>(33.3%)	3 XII (100%)	3 R (100%)	3 HC<br>(100%)	3 E (100%)	3 Endo-<br>scopic Tran-<br>soral (100%)	3 GTR<br>(100%)	1 Dysphagia<br>(33.3%)	0 (0%)	3 (100%)	2 IX, X, XII<br>(66.7%)<br>1 XII (33.3%)	0 (0%)		6.0	3 Alive<br>(100%)
**Helms et al. – 2012[67]**	Case report – V	1 /<br>0 (0%)	29	0 (0%)	C1	L	FM-C1	I-E, ID-ED	FL + CL<br>(C1-C2)	GTR	0 (0%)	0 (0%)	1 (100%)	0 (0%)	0 (0%)		36.0	1 Alive<br>(100%)
**Nakamizo et al. – 2012[68]**	Case report – V	1 /<br>1 (100%)	49	0 (0%)	C2	R	C1-C2 F	E, ED	CL (C1-C2)	GTR	0 (0%)	0 (0%)	1 (100%)	0 (0%)	0 (0%)		3.0	1 Alive<br>(100%)
**Ohba et al. – 2012[69]**	Case report – V	1 /<br>1 (100%)	37	V, IX, X, XI, XII	XII	L	HC	I-E, ID-ED	SC	PTR	0 (0%)	0 (0%)	1 (100%)	V, IX, X, XI,<br>XII	1 (100%)	24.0	108.0	1 Alive<br>(100%)

**Oyama et al. – 2012[70]**	Case report – V	1 / 1 (100%)	25	XII	XII	R	HC	I-E, ID-ED	TC	PTR	Dysphagia	1 (100%)	0 (0%)	XII	0 (0%)	25.0	37.0	1 Alive (100%)
**Santarius et al. – 2012[71]**	Case report – V	1 / 0 (0%)	9	VII	XII	L	HC	I-E, ID-ED	SC	GTR	Hemiparesis	0 (0%)	1 (100%)	XII	1 (100%)		12.0	1 Alive (100%)
**Wan et al. – 2012[72]**	Case series – IV	8 / 4 (50%)	49 (31-58)	4 VII, VIII, IX, X, XI (50%)<br>2 IX, X, XII (25%)<br>1 X (12.5%)<br>1 VIII, IX, XII (12.5%)	8 IX-X-XI (100%)	N/A	8 JF (100%)	8 I-E, ID-ED (100%)	8 RS-SJ (100%)	7 GTR (87.5%)<br>1 PTR (12.5%)	2 CSF leak (25%)<br>1 Aspiration pneumonia (12.5%)<br>1 Dysphagia (12.5%)	1 (12.5%)	8 (100%)	4 VII, VIII, IX, X, XI (50%)<br>2 IX, X, XII (25%)<br>1 X (12.5%)<br>1 VIII, IX, XII (12.5%)	0 (0%)		38 (23-60)	1 Alive (100%)
**Zhang et al. – 2012[73]**	Case series – IV	3 / 0 (0%)	42 (16-43)	1 X, XII (33.3%)<br>1 VII, VIII, XII (33.3%)	3 XII (100%)	2 L (66.7%)<br>1 R (33.3%)	JF & HC	3 I-E, ID-ED (100%)	3 Endo-scopic Tran-soral (100%)	3 GTR (100%)	0 (0%)	0 (0%)	3 (100%)	1 X (33.3%)<br>1 XII (33.3%)	0 (0%)		4.0 (2-6)	3 Alive (100%)
**Baghel et al. – 2013[74]**	Case report – V	1 / 1 (100%)	40	XII	XII	L	HC	I-E, ID-ED	SC-RS + CL (C1)	PTR	0 (0%)	0 (0%)	1 (100%)	XII	0 (0%)		2.0	1 Alive (100%)
**Chowdhury et al. – 2013[75]**	Case series – IV	14 / 7 (50%)	31 (10-53)	0 (0%)	11 C1 (84.6%)<br>2 C2 (15.4%)	8 L (57.1%)<br>6 R (43.9%)	11 FM-C1 (84.6%)<br>2 C1-C2 F (15.4%)	6 E, ID-ED (46.2%)<br>5 E, ID (38.5%)<br>3 E, ED (23.1%)	14 SC + CL (C1-C2 (100%)	14 GTR (100%)	2 CSF leak (15.4%)<br>1 Aspiration pneumonia (7.1%)	0 (0%)	14 (100%)	0 (0%)	0 (0%)		8.0 (3-12)	13 Alive (92.9%)<br>1 Dead (7.1%)
**Xiao et al. – 2013[76]**	Case series – IV	11 / 3 (27.3%)	50 (31-59)	6 IX, XII (54.5%)<br>2 VII, IX, XII (18.2%)<br>2 VIII, IX, X, XII (18.2%)<br>1 XII (9.1%)	11 XI (100%)	7 R (63.6%)<br>4 L (36.4%)	11 HC (100%)	11 I-E (100%)	11 FL (100%)	10 GTR (90.9%)<br>1 PTR (9.1%)	2 Pneumonia (18.2%)<br>1 Meningitis (9.1%)	0 (0%)	11 (100%)	N/A	0 (0%)		14 (3-25)	11 Alive (100%)
**Nowak et al. – 2014[77]**	Case series – IV	10 / 6 (60%)	38 (25-54)	2 IX, X, XI (20%)<br>2 VII, VIII, IX, X (20%)<br>1 VIII (10%)<br>1 XI (10%)<br>1 V, VII, VIII, IX (10%)<br>1 IX, X, XI, XII (10%)<br>1 VII, VIII, IX, X, XI (10%)	5 IX-X-XI (50%)<br>3 IX (30%)<br>1 X (10%)<br>1 XI (10%)	N/A	10 JF (100%)	8 I, ED (80%)<br>2 I, ID (20%)	8 SC-CL (C1) (80%)<br>2 RS (20%)	10 GTR (100%)	6 Paresis (60%)	0 (0%)	4 (40%)	5 VIII (50%)<br>5 X (50%)<br>4 XI (40%)<br>3 VII (30%)<br>3 IX (30%)<br>1 V (10%)	1 (10%)	40	28 (6-71)	10 Alive (100%)
**Suri et al. – 2014[78]**	Case series – IV	14 / 8 (57.1%)	35 (15-63)	14 XII (100%)<br>8 IX, X (57.1%)	14 XII (100%)	9 R (69.2%)<br>4 L (30.8%)<br>1 B (7.1%)	14 HC (100%)	13 I, ID (92.9%)<br>1 I, ID-ED (7.1%)	9 RS (64.3%)<br>3 SC (21.4%)<br>2 FL (14.3%)	9 PTR (64.3%)<br>5 GTR (35.7%)	2 Infection wound (14.3%)<br>1 CSF leak (7.1%)	7 (50%)	14 (100%)	14 XII (100%)	0 (0%)		32 (3-59)	14 Alive (100%)
**Yu et al. – 2014[79]**	Case series – IV	9 / 5 (55.6%)	52 (21-69)	0 (0%)	9 C2 (100%)	5 R (55.6%)<br>4 L (44.4%)	9 C1-C2 F (100%)	8 E, ID-ED (88.9%)<br>1 E, ED (11.1%)	9 SC + CL (C1-C2) (100%)	9 GTR (100%)	2 C2 deficit (22.2%)	0 (0%)	9 (100%)	0 (0%)	0 (0%)		34 (5-72)	9 Alive (100%)
**Feldman et al. – 2015[80]**	Case report – V	1 / 1 (100%)	64	0 (0%)	C2	R	C1-C2 F	E, ID-ED	FL + CL (C1-C2)	GTR	0 (0%)	0 (0%)	1 (100%)	0 (0%)	1 (100%)	12.0	36.0	1 Alive (100%)
**Hayashi et al. – 2015[81]**	Case series – IV	8 / 5 (62.5%)	44 (38-72)	4 XII (50%)<br>3 X, XII (37.5%)<br>1 IX, X, XII (12.5%)	8 XII (100%)	5 L (62.5%)<br>3 R (37.5%)	8 HC (100%)	8 I, ID (100%)	6 TC (75%)<br>2 SC (25%)	8 PTR (100%)	0 (0%)	0 (0%)	8 (100%)	N/A	0 (0%)		6.0 (3-12)	1 Alive (100%)
**Jiang et al. – 2015[82]**	Case series – IV	6 / 5 (83.3%)	54 (37-72)	0 (0%)	5 C1 (83.3%)<br>1 C2 (16.7%)	N/A	6 FM-C2 (100%)	6 E (100%)	6 CL (C1) (100%)	6 GTR (100%)	2 CSF leak (33.3%)	0 (0%)	5 (83.3%)	0 (0%)	0 (0%)		33.5 (24-42)	1 Alive (100%)
**Oichi et al. – 2015[83]**	Case report – V	1 / 1 (100%)	64	0 (0%)	C2	L	C1-C2 F	E, ED	CL (C1)	GTR	0 (0%)	0 (0%)	1 (100%)	0 (0%)	0 (0%)		6.0	1 Alive (100%)
**Puchalska-Niedbal et al. – 2015[84]**	Case report – V	1 / 0 (0%)	24	0 (0%)	C1	N/A	FM-C1	E, ID	SC + CL (C1-C2)	PTR	0 (0%)	0 (0%)	1 (100%)	0 (0%)	0 (0%)		2.0	1 Alive (100%)
**Yu et al. – 2015[85]**	Case report – V	1 / 1 (100%)	61	XII	XII	L	HC	I-E, ID-ED	FL-SC	GTR	0 (0%)	0 (0%)	1 (100%)	XII	0 (0%)		24.0	1 Alive (100%)
**Bhatoe et al. – 2016[86]**	Case report – V	1 /	47	IX, X, XII	XII	L	HC	I, E, ID	RS	GTR	0 (0%)	0 (0%)	1 (100%)	XII	0 (0%)		6.0	1 Alive

		0 (0%)															(100%)
**Liebelt et al. – 2016[87]**	Case report – V	1 / 1 (100%)	58	0 (0%)	C1	R	FM-C1	I-E, ID-ED	SC + CL (C1)	PTR	0 (0%)	0 (0%)	0 (0%)	0 (0%)	0 (0%)	3.0	1 Alive (100%)
**Yoon et al. – 2016[88]**	Case report – V	1 / 0 (0%)	32	0 (0%)	C2	L	C1-C2 F	I-E, ID-ED	SC + CL (C1)	GTR	0 (0%)	0 (0%)	0 (0%)	0 (0%)	0 (0%)	1.5	1 Alive (100%)
**Inoue et al. – 2017[89]**	Case report – V	1 / 1 (100%)	61	XII	XII	R	HC	I-E, ID-ED	SC-RS	GTR	Hoarse, dys-phagia	0 (0%)	0 (0%)	XII	0 (0%)	12.0	1 Alive (100%)
**Lee et al. – 2017[90]**	Case report – V	1 / 1 (100%)	57	0 (0%)	C2	L	C1-C2 F	E, ED	CL (C2-C3)	GTR	SAH	0 (0%)	0 (0%)	0 (0%)	0 (0%)	2.0	1 Alive (100%)
**Pisani et al. – 2017[91]**	Case report – V	1 / 0 (0%)	37	0 (0%)	C1	L	FM-C1	E, ED	SC	GTR	0 (0%)	0 (0%)	1 (100%)	0 (0%)	0 (0%)	6.0	1 Alive (100%)
**Goel et al. – 2018[92]**	Case series – IV	50 / 36 (72%)	36 (14-70)	0 (0%)	50 C2 (100%)	5 B (10%)	50 C1-C2 (100%)	50 E, ID (100%)	50 CL (C1-C3) (100%)	50 PTR (100%)	N/A	0 (0%)	50 (100%)	0 (0%)	0 (0%)	68 (3-120)	50 Alive (100%)
**Heda et al. – 2018[93]**	Case report – V	1 / 0 (0%)	40	XII	XII	L	JF & HC	E, ED	SC-RS	PTR	0 (0%)	0 (0%)	1 (100%)	XII	0 (0%)	3.0	1 Alive (100%)
**Matsuda et al. – 2018[94]**	Case report – IV	2 / 2 (100%)	61.5 (53-70)	0 (0%)	2 C2 (100%)	2 R (100%)	C1-C2 F	1 E, ED (50%)<br>1 E, ID-ED (50%)	1 CL (C1-C2 (50%)<br>1SC+CL (C1-C2) (50%)	2 GTR (100%)	0 (0%)	0 (0%)	0 (0%)	0 (0%)	0 (0%)	3.0	2 Alive (100%)
**Park et al. – 2018[95]**	Case report – V	1 / 1 (100%)	54	0 (0%)	C2	R	C1-C2 F	E, ID	CL (C1)	PTR	0 (0%)	0 (0%)	1 (100%)	0 (0%)	0 (0%)	12.0	1 Alive (100%)
**Jung et al. – 2019[96]**	Case report – V	1 / 1 (100%)	37	0 (0%)	C2	R	C1-C2 F	E, ID	CL (C2-C3)	GTR	0 (0%)	0 (0%)	1 (100%)	0 (0%)	0 (0%)	1.0	1 Alive (100%)
**Di Pascuale et al. – 2020[97]**	Case report – V	1 / 1 (100%)	22	VIII, IX, X, XII	XII	L	HC	I-E, ED	RS	PTR	Dysphagia	0 (0%)	1 (100%)	0 (0%)	0 (0%)	12.0	1 Alive (100%)
**Nakajima et al. – 2020[98]**	Case report – V	1 / 0 (0%)	50	0 (0%)	C2	R	C1-C2 F	E, ED	CL (C1-C2)	PTR	0 (0%)	0 (0%)	1 (100%)	0 (0%)	0 (0%)	36.0	1 Alive (100%)
**Aftahy et al. – 2021[99]**	Case series – IV	9 / 2 (22.2%)	43 (20-71)	1 VII (11.1%)<br>1 VIII (11.1%)<br>1 X (11.1%)	5 IX (55.6%)<br>2 X (22.2%)<br>2 IX-X-XI (22.2%)	N/A	9 JF (100%)	4 I, ED (44.4%)<br>4 I (44.4%)<br>1 E (11.1%)	6 RS (66.7%)<br>3 ELITE (33.3%)	9 GTR (100%)	2Hydrocepha-lus (22.2%)<br>2 Hoarse, hear loss (22.2%)	0 (0%)	9 (100%)	N/A	0 (0%)	16.5 (3-84)	9 Alive (100%)
**Fachniadin et al. – 2021[100]**	Case report – V	1 / 0 (0%)	53	IX, X, XII	XII	L	JF & HC	I, ID	SC-RS	PTR	0 (0%)	0 (0%)	1 (100%)	XI, XII	0 (0%)	1.0	1 Alive (100%)
**Matsui et al. – 2021[101]**	Case report – V	1 / 0 (0%)	54	0 (0%)	C1	R	FM-C1	I-E, ID-ED	SC + CL (C1)	PTR	0 (0%)	0 (0%)	1 (100%)	0 (0%)	0 (0%)	3.0	1 Alive (100%)

References correspond to **Supplementary File S1.**

Abbreviations: **B**, Bilateral; **CL**, Cervical laminectomy/laminoplasty; **E**, Extracranial; **ED**, extradural; **ELITE**, Extreme lateral infrajugular transcondylar-transtubercular exposure; **F**, Foramen; **FL**, Far-lateral; **FM**, Foramen magnum; **GTR**, Gross total resection (100%); **HC**, Hypoglossal canal; **I**, Intracranial; **ID**, Intradural; **JF**, Jugular foramen; **L**, Left; **M**, Midline; **N/A**, Not Available; **OS**, Overall Survival; **PFS**, Progression-free survival; **PTR**, Partial resection (<100%); **R**, Right; **RS**, Retrosigmoid; **SAH**, Subarachnoid hemorrhage; **SC**, Suboccipital craniectomy; **SJ**, Suprajugular; **TC**, Transcondylar.

References of included articles.

1. Scott M, Wycis HT. Intracranial Neurinoma of the Hypoglossal Nerve. *J Neurosurg*. 1949;6(4):333-336. doi:10.3171/jns.1949.6.4.0333
2. Morelli RJ. Intracranial neurilemmoma of the hypoglossal nerve: Review and case report. *Neurology*. 1966;16(7):709-709. doi:10.1212/WNL.16.7.709
3. Strang RR. Intracranial Neurinoma of the Hypoglossal Nerve. *ANZ J Surg*. 1967;37(2):187-189. doi:10.1111/j.1445-2197.1967.tb04009.x
4. Bartal AD, Djaldetti MM, Mandel EM, Lerner MA. Dumb-bell neurinoma of the hypoglossal nerve. *J Neurol Neurosurg Psychiatry*. 1973;36(4):592-595. doi:10.1136/jnnp.36.4.592
5. Sumra RS, Hölper G, Wanis A. Intramedullary syndrome due to an extradural neurinoma near the foramen magnum. *J Neurol*. 1978;218(2):145-148. doi:10.1007/BF02402174
6. Conrad B, Mergner T. High cervical neurinoma (C1/C2) diagnosed falsely as multiple sclerosis because of trigeminal neuralgia. *Arch f�r Psychiatr und Nervenkrankheiten*. 1979;227(1):33-37. doi:10.1007/BF00585675
7. Fujiwara S, Hachisuga S, Numaguchi Y. Intracranial hypoglossal neurinoma: Report of a case. *Neuroradiology*. 1980;20(2):87-90. doi:10.1007/BF00339552
8. Ulsø C, Sehested P, Overgaard J. Intracranial hypoglossal neurinoma: Diagnosis and postoperative care. *Surg Neurol*. 1981;16(1):65-68. doi:10.1016/S0090-3019(81)80070-5
9. Berger MS, Edwards MSB, Bingham WG. Hypoglossal Neurilemoma. *Neurosurgery*. 1982;10(5):617-620. doi:10.1227/00006123-198205000-00013
10. Dolan EJ, Tucker WS, Rotenberg D, Chui M. Intracranial hypoglossal schwannoma as an unusual cause of facial nerve palsy. *J Neurosurg*. 1982;56(3):420-423. doi:10.3171/jns.1982.56.3.0420

11. Hakuba A, Komiyama M, Tsujimoto T, et al. Transuncodiscal approach to dumbbell tumors of the cervical spinal canal. *J Neurosurg*. 1984;61(6):1100-1106. doi:10.3171/jns.1984.61.6.1100

12. Tuck RR, Mokri B, Cilluffo JM. Intracranial Schwannoma of the Hypoglossal Nerve. *Arch Neurol*. 1984;41(5):502-505. doi:10.1001/archneur.1984.04050170048015

13. Bettoni L, Baldi P, Bertolino G. CT diagnosis of intracranial neurinoma of the hypoglossal nerve. *J Comput Assist Tomogr*. 9(4):827-828. doi:10.1097/00004728-198507010-00037

14. Lesoin F, Rousseaux M, Martin HJ, Petit H, Jomin M. Astereognosis and amyotrophy of the hand with neurinoma of the second cervical nerve root. *J Neurol*. 1986;233(1):57-58. doi:10.1007/BF00313995

15. Shearman CP, Pearman K, Hockley AD. Dumb-bell schwannoma of the jugular foramen. *Child's Nerv Syst*. 1986;2(1):52-53. doi:10.1007/BF00274037

16. Solymosi L, Wassmann H, Bonse R. Diagnosis of neurinoma in the region of the jugular foramen — a case report. *Neurosurg Rev*. 1987;10(1):41-46. doi:10.1007/BF01780593

17. Nakashima T, Tomita K, Uemura T, Matsushima T, Fukui M. Accessory nerve schwannoma extending extracranially to the second cervical level of vertebra. *J Laryngol Otol*. 1988;102(10):959-961. doi:10.1017/S0022215100106929

18. Crockard HA, Sen CN. The transoral approach for the management of intradural lesions at the craniovertebral junction. *Neurosurgery*. 1991;9(4):88. doi:10.1097/00006123-199101000-00014

19. Kratimenos GP, Crockard HA. The far lateral approach for ventrally placed foramen magnum and upper cervical spine tumours. *Br J Neurosurg*. 1993;7(2):129-140. doi:10.3109/02688699309103469

20. Krauss J, Bertalanffy H, Schwechheimer K, Müller A, Seeger W. Ventral brain stem schwannoma at the entry zone of the hypoglossal nerve. *min - Minim Invasive Neurosurg*. 1993;36(02):66-69. doi:10.1055/s-2008-1053798

21. Kamitani H, Masuzawa H, Kanazawa I, Kubo T, Tokuyama Y. A combined extradural-posterior petrous and suboccipital approach to the jugular foramen tumours. *Acta Neurochir (Wien)*. 1994;126(2-4):179-184. doi:10.1007/BF01476430

22. Okura A, Shigemori M, Abe T, Yamashita M, Kojima K, Noguchi S. Hemiatrophy of the tongue due to hypoglossal schwannoma shown by MRI. *Neuroradiology*. 1994;36(3):239-240. doi:10.1007/BF00588142

23. Smith PG, Backer RJ, Kletzker GR, et al. Surgical management of transcranial hypoglossal schwannomas. *Am J Otol*. 1995;16(4):451-456. http://www.ncbi.nlm.nih.gov/pubmed/8588644

24. Kobayashi S, Otsuka A, Tsunoda T, Inoue HK. Intracranial hypoglossal neurinoma without preoperative hypoglossal nerve paresis--case report. *Neurol Med Chir (Tokyo)*. 1996;36(6):384-387. doi:10.2176/nmc.36.384

25. Sato M, Kanai N, Fukushima Y, et al. Hypoglossal neurinoma extending intra- and extracranially: case report. *Surg Neurol*. 1996;45(2):172-175. doi:10.1016/s0090-3019(96)80012-7

26. Sairyo K, Henmi T, Endo H. Foramen magnum schwannoma with an unusual clinical presentation: case report. *Spinal Cord*. 1997;35(8):554-556. doi:10.1038/sj.sc.3100431

27. Bunc G, Milojković V, Košir G, Kovačič S, Rebol J. Dumb-bell Hypoglossal Neurinoma with Intra- and Extracranial Paravertebral Expansion. *Acta Neurochir (Wien)*. 1998;140(11):1209-1210. doi:10.1007/s007010050240

28. Gilsbach JM, Sure U, Mann W. The supracondylar approach to the jugular tubercle and hypoglossal canal. *Surg Neurol*. 1998;50(6):563-570.

doi:10.1016/S0090-3019(97)00378-9

29. Spinnato S, Talacchi A, Musumeci A, Turazzi S, Bricolo A. Dumbbell-Shaped Hypoglossal Neurinoma: Surgical Removal via a Dorsolateral Transcondylar Approach. A Case Report and Review of the Literature. *Acta Neurochir (Wien)*. 1998;140(8):827-832. doi:10.1007/s007010050185

30. Kachhara R, Nair S, Radhakrishnan V V. Large dumbbell neurinoma of hypoglossal nerve: case report. *Br J Neurosurg*. 1999;13(3):338-340. doi:10.1080/02688699943826

31. Hoshi M, Yoshida K, Ogawa K, Kawase T. Hypoglossal neurinoma--two case reports. *Neurol Med Chir (Tokyo)*. 2000;40(9):489-493. doi:10.2176/nmc.40.489

32. Kalavakonda C, Sekhar LN, Jones RV, Rehaman AB. Intermittent vertebral artery compression caused by C1-root schwannoma: Case Report. *Neurol Res*. 2000;22(7):679-684. doi:10.1080/01616412.2000.11740739

33. Mariniello G, Horvat A, Popovic M, Dolenc V V. Cellular dumbbell schwannoma of the hypoglossal nerve presenting without hypoglossal nerve palsy. *Clin Neurol Neurosurg*. 2000;102(1):40-43. doi:10.1016/S0303-8467(99)00079-7

34. Kyoshima K, Uehara T, Koyama J, Idomari K, Yomo S. Dumbbell C2 Schwannomas Involving Both Sensory and Motor Rootlets: Report of Two Cases. *Neurosurgery*. 2003;53(2):436-440. doi:10.1227/01.NEU.0000073992.97761.88

35. Leal Filho MB, Borges G, Ferreira A, França D, Mello P. Schwannoma of the craniocervical junction: surgical approach of two cases. *Arq Neuropsiquiatr*. 2003;61(3A):639-641. doi:10.1590/S0004-282X2003000400021

36. Passacantilli E, Lanzino G, Henn JS, Partovi S, Spetzler RF. Intracranial extradural schwannoma of the 12th cranial nerve. *J Neurosurg*. 2003;98(1):219. doi:10.3171/jns.2003.98.1.0219

37. Tatebayashi K, Tanaka Y, Numata H, Kawakami S, Kamitani H, Watanabe T. Schwannoma of the spinal accessory nerve in the cisterna magna. *Surg Neurol*. 2003;59(3):217-222. doi:10.1016/S0090-3019(02)01035-2

38. Aihara K, Morita A. Dumbbell-shaped hypoglossal schwannoma in an elderly woman: a clinical dilemma. *Surg Neurol*. 2005;63(6):526-528. doi:10.1016/j.surneu.2004.07.045

39. Ho CL, Deruytter MJ. Navigated dorsolateral suboccipital transcondylar (NADOSTA) approach for treatment of hypoglossal schwannoma. *Clin Neurol Neurosurg*. 2005;107(3):236-242. doi:10.1016/j.clineuro.2004.05.004

40. Kadri PAS, Al-Mefty O. Surgical treatment of dumbbell-shaped jugular foramen schwannomas. *Neurosurg Focus*. 2004;17(2):56-62. doi:10.3171/foc.2004.17.2.9

41. Kim JH, Lee JH, Park YK, Kwon TH, Chung HS. C-1 Root Schwannoma with Aggressive Lateral Mass Invasion. *Yonsei Med J*. 2005;46(4):575. doi:10.3349/ymj.2005.46.4.575

42. Takumi I, Isayama K, Osamura RY, Kobayashi S, Teramoto A. Intracisternal neurinoma of the C1 posterior root. *Acta Neurochir (Wien)*. 2005;147(11):1189-1192. doi:10.1007/s00701-005-0618-z

43. Sato K, Shimizu S, Oka H, Nakahara K, Utsuki S, Fujii K. Usefulness of transcervical approach for surgical treatment of hypoglossal schwannoma with paraspinal extension: case report. *Surg Neurol*. 2006;65(4):397-401. doi:10.1016/j.surneu.2005.08.020

44. Ueda R, Yoshida K, Kawase T. Intradural C-1 ventral root schwannomas treated by surgical resection via the lateral suboccipital transcondylar approach--three case reports. *Neurol Med Chir (Tokyo)*. 2006;46(6):298-301. doi:10.2176/nmc.46.298

45. Kikkawa Y, Shono T, Mizoguchi M, Nagata S, Sasaki T. A Case of Intracranial Hypoglossal Neurinoma Without Hypoglossal Nerve Palsy: Operative

View of the Preserved Rostral Trunk. *min - Minim Invasive Neurosurg*. 2007;50(5):296-299. doi:10.1055/s-2007-984385

46. Kim Y, Ko Y, Yi H, Oh SJ. A Case of Hypoglossal Neurilemmoma Resected Via Burr-hole Craniectomy. *J Korean Neurosurg Soc*. 2007;41:43-46. https://www.jkns.or.kr/journal/view.php?number=1784

47. Tucker A, Miyake H, Tsuji M, Ukita T, Nishihara K, Ohmura T. Intradural Microsurgery and Extradural Gamma Knife Surgery for Hypoglossal Schwannoma: Case Report and Review of the Literature. *min - Minim Invasive Neurosurg*. 2007;50(6):374-378. doi:10.1055/s-2007-993206

48. Ciappetta P, Colamaria A. Giant craniovertebral junction hemorrhagic schwannoma. *Neurosurgery*. 2008;62(5). doi:10.1227/01.NEU.0000316423.20361.5F

49. Garg N, Sampath S. Fatal delayed post-operative cerebral venous thrombosis after excision of hypoglossal nerve schwanomma. *Acta Neurochir (Wien)*. 2008;150(6):605-609. doi:10.1007/s00701-008-1498-1

50. Kuo L-T, Huang AP-H, Kuo K-T, Tseng H-M. Extradural dumbbell schwannoma of the hypoglossal nerve: a case report with review of the literature. *Surg Neurol*. 2008;70(SUPPL. 1):S34-S38. doi:10.1016/j.surneu.2007.11.023

51. Chibbaro S, Mirone G, Makiese O, Bresson D, George B. Dumbbell-shaped jugular foramen schwannomas: surgical management, outcome and complications on a series of 16 patients. *Neurosurg Rev*. 2009;32(2):151-159. doi:10.1007/s10143-009-0188-2

52. Heiroth H-J, Riemenschneider MJ, Steiger H-J, Hänggi D. A cylindrical extracranial cranial base neurinoma of the hypoglossal nerve: a rare tumor with a rare localization: case report. *Neurosurgery*. 2009;65(1):E212-3; discussion E213. doi:10.1227/01.NEU.0000347006.02222.CB

53. Ichimura S, Yoshida K, Kawase T. Surgical approach for hypoglossal schwannomas to prevent deformity of the atlanto-occipital joint. *Acta Neurochir (Wien)*. 2009;151(6):575-579. doi:10.1007/s00701-009-0284-7

54. Mathiesen T, Svensson M, Lundgren J, Kihlström L, Parisotto R, Bagger-Sjöbäck D. Hypoglossal schwannoma—successful reinnervation and functional recovery of the tongue following tumour removal and nerve grafting. *Acta Neurochir (Wien)*. 2009;151(7):837-841. doi:10.1007/s00701-009-0226-4

55. Watanabe M, Sakai D, Yamamoto Y, Iwashina T, Sato M, Mochida J. Upper cervical spinal cord tumors: review of 13 cases. *J Orthop Sci*. 2009;14(2):175-181. doi:10.1007/s00776-008-1309-4

56. Edizer DT, Mercan H, Cansiz H. Hypoglossal Schwannoma Presenting Only With Headache. *J Craniofac Surg*. 2010;21(1):261-262. doi:10.1097/SCS.0b013e3181c5a37c

57. Ishikawa M, Kusaka G, Takashima K, Kamochi H, Shinoda S. Intraoperative monitoring during surgery for hypoglossal schwannoma. *J Clin Neurosci*. 2010;17(8):1053-1056. doi:10.1016/j.jocn.2009.09.040

58. Ji C, Ahn J-G, Huh H-Y, Park C-K. Cervical Schwannoma Presenting with Acute Intracranial Subarachnoid Hemorrhage. *J Korean Neurosurg Soc*. 2010;47(2):137. doi:10.3340/jkns.2010.47.2.137

59. Li W, Hong X, Wang L, Ge P, Fu S, Luo Y. Large Cystic Hypoglossal Schwannoma with Fluid-Fluid Level: A Case Report. *Skull Base*. 2010;20(03):193-197. doi:10.1055/s-0029-1246219

60. Cavalcanti DD, Martirosyan NL, Verma K, et al. Surgical management and outcome of schwannomas in the craniocervical region. *J Neurosurg*. 2011;114(5):1257-1267. doi:10.3171/2010.5.JNS0966

61. Takami T, Yamagata T, Chokyu I, Ikeda H, Tsuyuguchi N, Ohata K. Surgery of spinal nerve sheath tumors originating from C1 or C2 of high cervical spine. *Neurol Med Chir (Tokyo)*. 2010;50(11):1044-1049. doi:10.2176/nmc.50.1044

62. Gopalakrishnan CV, Baldawa S, Neelima R, Nair S. Giant Cystic Craniovertebral Schwannoma Arising From C1 Dorsal Root Masquerading as a Fourth

Ventricular Lesion. *Neurosurg Q*. 2011;21(1):60-62. doi:10.1097/WNQ.0b013e3182086a16

63. Ramzan A, Malik KN, Makhdoomi R, Wani A, Jain A. Giant craniocervical schwannoma in a teenage girl. *Child's Nerv Syst*. 2011;27(9):1503-1506. doi:10.1007/s00381-011-1510-1

64. Nonaka Y, Grossi PM, Bulsara KR, Taniguchi RM, Friedman AH, Fukushima T. Microsurgical Management of Hypoglossal Schwannomas Over 3 Decades. *Oper Neurosurg*. 2011;69(SUPPL. 2):ons121-ons140. doi:10.1227/NEU.0b013e31822a547b

65. Wang J, Ou S-W, Wang Y-J, Wu A-H, Wu P-F, Wang Y-B. Microsurgical management of dumbbell C1 and C2 schwannomas via the far lateral approach. *J Clin Neurosci*. 2011;18(2):241-246. doi:10.1016/j.jocn.2010.03.048

66. Zhang Q, Lv H, Wang Z, Guo H, Li M. Endoscopic Transoral Approach for Extracranial Hypoglossal Schwannoma. *ORL*. 2011;73(5):282-286. doi:10.1159/000330270

67. Helms J, Michael L. Large Dumbbell-Shaped C1 Schwannoma Presenting as a Foramen Magnum Mass. *J Neurol Surg Reports*. 2012;73(01):032-036. doi:10.1055/s-0032-1311757

68. Nakamizo A, Suzuki SO, Shimogawa T, et al. Concurrent spinal nerve root schwannoma and meningioma mimicking single-component schwannoma. *Neuropathology*. 2012;32(2):190-195. doi:10.1111/j.1440-1789.2011.01239.x

69. Ohba S, Inaba M, Kinoshita Y, Nakagawa T, Shimizu K, Murakami H. Hypoglossal nerve schwannoma with intratumoral hemorrhage. *Brain Tumor Pathol*. 2012;29(1):43-46. doi:10.1007/s10014-011-0060-2

70. Oyama H, Kito A, Maki H, Hattori K, Noda T, Wada K. Schwannoma originating from lower cranial nerves: report of 4 cases. *Nagoya J Med Sci*. 2012;74(1-2):199-206. doi:10.18999/nagjms.74.1-2.199

71. Santarius T, Dakoji S, Afshari FT, et al. Isolated hypoglossal schwannoma in a 9-year-old child. *J Neurosurg Pediatr*. 2012;10(2):130-133. doi:10.3171/2012.3.PEDS11555

72. Wan J-H, Wu Y-H, Li Z-J, et al. Triple dumbbell-shaped jugular foramen schwannomas. *J Cranio-Maxillofacial Surg*. 2012;40(4):354-361. doi:10.1016/j.jcms.2011.05.012

73. Zhang Q, Kong F, Guo H, et al. Surgical treatment of dumbbell-shaped hypoglossal schwannoma via a pure endoscopic transoral approach. *Acta Neurochir (Wien)*. 2012;154(2):267-275. doi:10.1007/s00701-011-1193-0

74. Baghel P, Gupta A, Tripathi V, Reddy D. Hypoglossal schwannoma presenting as hemi-atrophy of the tongue. *Neurol India*. 2013;61(3):324. doi:10.4103/0028-3886.115092

75. Chowdhury FH, Haque MR, Sarker MH. High cervical spinal schwannoma; microneurosurgical management: an experience of 15 cases. *Acta Neurol Taiwan*. 2013;22(2):59-66. http://www.ncbi.nlm.nih.gov/pubmed/24030037

76. Xiao X, Wu Z, Zhang L, et al. Using a modified far-lateral approach to remove hypoglossal neurilemmomas: notes on technique. *J Neurosurg*. 2013;118(3):657-668. doi:10.3171/2012.9.JNS12176

77. Nowak A, Dziedzic T, Czernicki T, Kunert P, Marchel A. Surgical treatment of jugular foramen schwannomas. *Neurol Neurochir Pol*. 2014;48(3):188-195. doi:10.1016/j.pjnns.2014.05.004

78. Suri A, Bansal S, Sharma B, et al. Management of Hypoglossal Schwannomas: Single Institutional Experience of 14 Cases. *J Neurol Surg Part B Skull Base*. 2014;75(03):159-164. doi:10.1055/s-0033-1356924

79. Yu Y, Hu F, Zhang X, Gu Y, Xie T, Ge J. Application of the Hemi-Semi-Laminectomy Approach in the Microsurgical Treatment of C2 Schwannomas. *J*

Spinal Disord Tech. 2014;27(6):E199-E204. doi:10.1097/BSD.0b013e318299f606

80. Feldman M, Kimmell KT, Replogle R. Resection of an Occipital-Cervical Junction Schwannoma through a modified minimally invasive approach: Technical Note. *Surg Neurol Int*. 2015;6(5):177. doi:10.4103/2152-7806.156566

81. Hayashi S, Takahashi S, Shidoh S, Yoshida K. Spatial Relationship between Hypoglossal Schwannoma and the Vertebral Artery Using the Far-Lateral Approach. *Neurol Med Chir (Tokyo)*. 2015;55(9):744-748. doi:10.2176/nmc.oa.2014-0354

82. Jiang H, He J, Zhan X, He M, Zong S, Xiao Z. Occipito-cervical fusion following gross total resection for the treatment of spinal extramedullary tumors in craniocervical junction: a retrospective case series. *World J Surg Oncol*. 2015;13(1):279. doi:10.1186/s12957-015-0689-0

83. Oichi T, Chikuda H, Morikawa T, et al. Concurrent spinal schwannoma and meningioma mimicking a single cervical dumbbell-shaped tumor: case report. *J Neurosurg Spine*. 2015;23(6):784-787. doi:10.3171/2015.3.SPINE141315

84. Puchalska-Niedbał L, Kulik U, Kojder K, Lubiński W, Rzewuska B, Kojder I. The ophthalmologic signs in case of C1 neurinoma without hydrocephalus – case report. *Pomeranian J Life Sci*. 2016;61(3):278-281. doi:10.21164/pomjlifesci.106

85. Yu Z, Zhao G, Zhao Z, Li Y, Xie G. Giant recurrent dumbbell-shaped hypoglossal schwannoma in an elderly male: A case report. *Oncol Lett*. 2016;11(1):283-286. doi:10.3892/ol.2015.3911

86. Bhatoe H. Excision of hypoglossal neurinoma by condyle sparing suboccipital keyhole approach. *Asian J Neurosurg*. 2017;12(1):120. doi:10.4103/1793-5482.144203

87. Liebelt BD, Haider AS, Steele WJ, Krishna C, Blacklock JB. Spinal Schwannoma and Meningioma Mimicking a Single Mass at the Craniocervical Junction Subsequent to Remote Radiation Therapy for Acne Vulgaris. *World Neurosurg*. 2016;93:484.e13-484.e16. doi:10.1016/j.wneu.2016.07.046

88. Yoon S, Park H, Lee K-S, Park SW, Hong C-K. Single-Stage Operation for Giant Schwannoma at the Craniocervical Junction with Minimal Laminectomy: A Case Report and Literature Review. *Korean J Spine*. 2016;13(3):173. doi:10.14245/kjs.2016.13.3.173

89. Inoue T, Nonaka Y, Hirai H, et al. Single-stage total resection of giant dumbbell-shaped hypoglossal schwannoma: a case report. *Acta Neurochir (Wien)*. 2018;160(4):727-730. doi:10.1007/s00701-017-3431-6

90. Lee SB, Rhim CH, Roh SW, Jeon SR, Rhim SC. Vertebral Artery Injury in C2-3 Epidural Schwannoma Resection: A Case Report and Literature Review. *Korean J Neurotrauma*. 2017;13(1):39. doi:10.13004/kjnt.2017.13.1.39

91. Pisani D, Camenzuli C, Psaila J, Božanić S, Calleja-Agius J. A locally destructive, completely asymptomatic, C1-root schwannoma with base of skull invasion: a case report. *J Surg Case Reports*. 2017;2017(2):1-5. doi:10.1093/jscr/rjx015

92. Goel A, Kaswa A, Shah A, Rai S, Gore S, Dharurkar P. Extraspinal-Interdural Surgical Approach for C2 Neurinomas—Report of an Experience with 50 Cases. *World Neurosurg*. 2018;110:575-582. doi:10.1016/j.wneu.2017.08.191

93. Heda S, Karthik DK, Rao ES, Deshpande A. Hypoglossal canal schwannoma causing isolated left 12th cranial nerve palsy. *BMJ Case Rep*. 2018;2018:bcr-2018-225544. doi:10.1136/bcr-2018-225544

94. Matsuda S, Kajihara Y, Abiko M, et al. Concurrent Schwannoma and Meningioma Arising in the Same Spinal Level: A Report of Two Cases. *NMC Case Rep J*. 2018;5(4):105-109. doi:10.2176/nmccrj.cr.2017-0207

95. Park JM, Kim JH. False localizing sign caused by schwannoma in cervical spinal canal at C1-2 level. *Medicine (Baltimore)*. 2018;97(36):e12215. doi:10.1097/MD.0000000000012215

96. Jung GS, Lee YM, Kim YZ, Kim JS. Intratumoral Hemorrhage of the Cervical Spinal Schwannoma Presenting: Acute Quadriparesis. *Brain Tumor Res*

Treat. 2019;7(2):160. doi:10.14791/btrt.2019.7.e34

97. Di Pascuale I, Brito N, Zerpa J, Roa C, Faria G. Hypoglossal Nerve Schwannoma: Case Report and Literature Review. *World Neurosurg*. 2020;135:205-208. doi:10.1016/j.wneu.2019.12.006

98. Nakajima R, Kinoshita M, Yahata T, Nakada M. Recovery time from supplementary motor area syndrome: relationship to postoperative day 7 paralysis and damage of the cingulum. *J Neurosurg*. 2020;132(3):865-874. doi:10.3171/2018.10.JNS182391

99. Aftahy AK, Groll M, Barz M, et al. Surgical Management of Jugular Foramen Schwannomas. *Cancers (Basel)*. 2021;13(16):4218. doi:10.3390/cancers13164218

100. Fachniadin A, Widi Nugroho S, Ananda Aman R, et al. A Dumbbell-shaped hypoglossal schwannoma managed by a combination of open surgery and endoscopic assistance. *Interdiscip Neurosurg*. 2021;25:101102. doi:10.1016/j.inat.2021.101102

101. Matsui Y, Fukumura M, Toda M. Cystic Dumbbell-Shaped C1 Schwannoma with Intracranial Extension and Hydrocephalus. *World Neurosurg*. 2021;148:24-28. doi:10.1016/j.wneu.2020.12.173
