# Supplementary material for: Surgical Management of Craniovertebral Junction Schwannomas: A Systematic Review
Source: Curr Oncol. 2022 Jul 9;29(7):4842–55. doi: 10.3390/curroncol29070384 (PMC9319499; doi:10.3390/curroncol29070384)
Supplement: Supplementary file 1 [file curroncol-29-00384-s001.zip › Supplementary File S2.pdf]

Supplementary File S2. Risk of bias assessments for included studies.

**Joanna Briggs Institute Checklist for Case Reports – Criteria**
1. Were patient's demographic characteristics clearly described?
2. Was the patient's history clearly described and presented as a timeline?
3. Was the current clinical condition of the patient on presentation clearly described?
4. Were diagnostic tests or assessment methods and the results clearly described?
5. Was the intervention(s) or treatment procedure(s) clearly described?
6. Was the post-intervention clinical condition clearly described?
7. Were adverse events (harms) or unanticipated events identified and described?
8. Does the case report provide takeaway lessons?
**Responses Options**: Yes, No, Unclear, Not Applicable (NA)
**Quality Rating**: Poor 0 – 2; Fair 3 – 5; Good 6 – 8

Study (Case Reports)	1	2	3	4	5	6	7	8	Rating
Scott et al. – 1949[1]	Yes	Yes	Yes	Yes	Yes	Yes	Yes	No	7 – Good
Morelli et al. – 1966[2]	Yes	Yes	Yes	Yes	Yes	Yes	Yes	Yes	8 – Good
Strang et al. – 1967[3]	Yes	Yes	Yes	Yes	Yes	Yes	Yes	Yes	8 – Good
Bartal et al. – 1973[4]	Yes	Yes	Yes	Yes	Yes	Yes	Yes	Yes	8 – Good
Sumra et al. – 1978[5]	Yes	Yes	Yes	Yes	Yes	No	No	Yes	6 – Good
Conrad et al. – 1979[6]	Yes	Yes	Yes	Yes	Yes	No	No	Yes	6 – Good
Fujiwara et al. – 1980[7]	Yes	Yes	Yes	Yes	Yes	Yes	Yes	Yes	8 – Good
Ulsø et al. – 1981[8]	Yes	Yes	Yes	Yes	Yes	Yes	Yes	Yes	8 – Good
Berger et al. – 1982[9]	Yes	Yes	Yes	Yes	Yes	Yes	Yes	Yes	8 – Good
Dolan et al. – 1982[10]	Yes	Yes	Yes	Yes	Yes	Yes	Yes	Yes	8 – Good
Bettoni et al. – 1985[13]	Yes	Yes	Yes	Yes	Yes	Yes	Yes	Yes	8 – Good
Lesoin et al. – 1986[14]	Yes	Yes	Yes	Yes	Yes	Yes	Yes	Yes	8 – Good
Shearman et al. – 1986[15]	Yes	Yes	Yes	Yes	Yes	Yes	Yes	Yes	8 – Good
Solymosi et al. – 1987[16]	Yes	Yes	Yes	Yes	Yes	No	No	Yes	6 – Good
Nakashima et al. – 1988[17]	Yes	Yes	Yes	Yes	Yes	No	No	Yes	6 – Good
Krauss et al. – 1993[20]	Yes	Yes	Yes	Yes	Yes	Yes	Yes	Yes	8 – Good
Okura et al. – 1994[22]	Yes	Yes	Yes	Yes	Yes	Yes	Yes	Yes	8 – Good
Smith et al. – 1995[23]	Yes	Yes	Yes	Yes	Yes	No	No	Yes	6 – Good
Kobayashi et al. – 1996[24]	Yes	Yes	Yes	Yes	Yes	No	No	Yes	6 – Good
Sato et al. – 1996[25]	Yes	Yes	Yes	Yes	Yes	Yes	Yes	Yes	8 – Good
Sairyo et al. – 1997[26]	Yes	Yes	Yes	Yes	Yes	No	No	Yes	6 – Good
Bunc et al. – 1998[27]	Yes	Yes	Yes	Yes	Yes	Yes	Yes	Yes	8 – Good
Gilsback et al. – 1998[28]	Yes	Yes	Yes	Yes	Yes	Yes	Yes	Yes	8 – Good
Spinnato et al. – 1999[29]	Yes	Yes	Yes	Yes	Yes	No	No	Yes	6 – Good
Kachhara et al. – 1999[30]	Yes	Yes	Yes	Yes	Yes	No	No	Yes	6 – Good
Kalavakonda et al. – 2000[32]	Yes	Yes	Yes	Yes	Yes	Yes	Yes	Yes	8 – Good

Mariniello et al. – 2000[33]	Yes	Yes	Yes	Yes	Yes	Yes	Yes	Yes	8 – Good
Passacantilli et al. – 2003[36]	Yes	Yes	Yes	Yes	Yes	Yes	Yes	No	7 – Good
Tatebayashi et al. – 2003[37]	Yes	Yes	Yes	Yes	Yes	Yes	Yes	Yes	8 – Good
Aihara et al. – 2004[38]	Yes	Yes	Yes	Yes	Yes	Yes	Yes	Yes	8 – Good
Ho et al. – 2004[39]	Yes	Yes	Yes	Yes	Yes	Yes	Yes	Yes	8 – Good
Kim et al. – 2005[41]	Yes	Yes	Yes	Yes	Yes	No	No	Yes	6 – Good
Takumi et al. – 2005[42]	Yes	Yes	Yes	Yes	Yes	Yes	Yes	Yes	8 – Good
Sato et al. – 2006[43]	Yes	Yes	Yes	Yes	Yes	Yes	Yes	Yes	8 – Good
Kikkawa et al. – 2007[45]	Yes	Yes	Yes	Yes	Yes	No	No	Yes	6 – Good
Kim et al. – 2007[46]	Yes	Yes	Yes	Yes	Yes	Yes	Yes	Yes	8 – Good
Tucker et al. – 2007[47]	Yes	Yes	Yes	Yes	Yes	Yes	Yes	Yes	8 – Good
Ciappetta et al. – 2008[48]	Yes	Yes	Yes	Yes	Yes	Yes	Yes	Yes	8 – Good
Garg et al. – 2008[49]	Yes	Yes	Yes	Yes	Yes	Yes	Yes	Yes	8 – Good
Kuo et al. – 2008[50]	Yes	Yes	Yes	Yes	Yes	Yes	Yes	Yes	8 – Good
Heiroth et al. – 2009[52]	Yes	Yes	Yes	Yes	Yes	Yes	Yes	Yes	8 – Good
Edizer et al. – 2010[56]	Yes	Yes	Yes	Yes	Yes	No	No	Yes	6 – Good
Ishikawa et al. – 2010[57]	Yes	Yes	Yes	Yes	Yes	Yes	Yes	Yes	8 – Good
Ji et al. – 2010[58]	Yes	Yes	Yes	Yes	Yes	No	No	Yes	6 – Good
Li et al. – 2010[59]	Yes	Yes	Yes	Yes	Yes	Yes	Yes	Yes	8 – Good
Gopalakrishnamet al. – 2011[62]	Yes	Yes	Yes	Yes	Yes	No	No	Yes	6 – Good
Ramzan et al. – 2011[63]	Yes	Yes	Yes	Yes	Yes	No	No	Yes	6 – Good
Helms et al. – 2012[67]	Yes	Yes	Yes	Yes	Yes	Yes	Yes	Yes	8 – Good
Nakamizo et al. – 2012[68]	Yes	Yes	Yes	Yes	Yes	No	No	Yes	6 – Good
Ohba et al. – 2012[69]	Yes	Yes	Yes	Yes	Yes	Yes	Yes	Yes	8 – Good
Oyama et al. – 2012[70]	Yes	Yes	Yes	Yes	Yes	Yes	Yes	Yes	8 – Good
Santarius et al. – 2012[71]	Yes	Yes	Yes	Yes	Yes	Yes	Yes	Yes	8 – Good
Baghel et al. – 2013[74]	Yes	Yes	Yes	Yes	Yes	No	No	Yes	6 – Good
Feldman et al. – 2015[80]	Yes	Yes	Yes	Yes	Yes	Yes	Yes	Yes	8 – Good
Oichi et al. – 2015[83]	Yes	Yes	Yes	Yes	Yes	Yes	Yes	Yes	8 – Good
Puchalska-Niedbał et al. – 2015[84]	Yes	Yes	Yes	Yes	Yes	Yes	Yes	Yes	8 – Good
Yu et al. – 2015[85]	Yes	Yes	Yes	Yes	Yes	Yes	Yes	Yes	8 – Good
Bhatoe et al. – 2016[86]	Yes	Yes	Yes	Yes	Yes	Yes	Yes	Yes	8 – Good
Liebelt et al. – 2016[87]	Yes	Yes	Yes	Yes	Yes	No	No	Yes	6 – Good
Yoon et al. – 2016[88]	Yes	Yes	Yes	Yes	Yes	No	No	Yes	6 – Good
Inoue et al. – 2017[89]	Yes	Yes	Yes	Yes	Yes	Yes	Yes	Yes	8 – Good
Lee et al. – 2017[90]	Yes	Yes	Yes	Yes	Yes	Yes	Yes	Yes	8 – Good
Pisani et al. – 2017[91]	Yes	Yes	Yes	Yes	Yes	Yes	Yes	Yes	8 – Good
Heda et al. – 2018[93]	Yes	Yes	Yes	Yes	Yes	Yes	No	Yes	7 – Good
Park et al. – 2018[95]	Yes	Yes	Yes	Yes	Yes	Yes	Yes	Yes	8 – Good
Jung et al. – 2019[96]	Yes	Yes	Yes	Yes	Yes	Yes	Yes	Yes	8 – Good
Di Pascuale et al. – 2020[97]	Yes	Yes	Yes	Yes	Yes	Yes	Yes	Yes	8 – Good
Nakajima et al. – 2020[98]	Yes	Yes	Yes	Yes	Yes	Yes	Yes	Yes	8 – Good
Fachniadin et al. – 2021[100]	Yes	Yes	Yes	Yes	Yes	Yes	Yes	Yes	8 – Good
Matsui et al. – 2021[101]	Yes	Yes	Yes	Yes	Yes	Yes	Yes	Yes	8 – Good

References correspond to Supplementary File S1.

**Joanna Briggs Institute Checklist for Case Series – Criteria**
1. Were there clear criteria for inclusion in the case series?
2. Was the condition measured in a standard, reliable way for all participants included in the case series?
3. Were valid methods used for identification of the condition for all participants included in the case series?
4. Did the case series have consecutive inclusion of participants?
5. Did the case series have complete inclusion of participants?
6. Was there clear reporting of the demographics of the participants in the study?
7. Was there clear reporting of clinical information of the participants?
8. Were the outcomes or follow up results of cases clearly reported?
9. Was there clear reporting of the presenting site(s)/clinic(s) demographic information?
10. Was statistical analysis appropriate?
**Responses Options**: Yes, No, Unclear, Not Applicable (NA)
**Quality Rating**: Poor 0 – 3; Fair 4 – 7; Good 8 – 10

**Study (Case Series)**	**1**	**2**	**3**	**4**	**5**	**6**	**7**	**8**	**9**	**10**	**Rating**
Hakuba et al. – 1984[11]	Yes	Yes	Yes	Yes	Yes	Yes	Yes	Yes	No	NA	8 – Good
Tuck et al. – 1984[12]	Yes	Yes	Yes	Yes	Yes	Yes	Yes	Yes	No	NA	8 – Good
Crockard et al. – 1991[18]	Yes	Yes	Yes	Yes	Yes	Yes	Yes	Yes	No	NA	8 – Good
Kratimenos et al. – 1993[19]	Yes	Yes	Yes	Yes	Yes	Yes	Yes	Yes	No	NA	8 – Good
Kamitani et al. – 1994[21]	Yes	Yes	Yes	Yes	Yes	Yes	Yes	Yes	No	NA	8 – Good
Hoshi et al. – 2000[31]	Yes	Yes	Yes	Yes	Yes	Yes	Yes	Yes	No	NA	8 – Good
Kyoshima et al. – 2003[34]	Yes	Yes	Yes	Yes	Yes	Yes	Yes	Yes	No	NA	8 – Good
Leal Filho et al. – 2003[35]	Yes	Yes	Yes	Yes	Yes	Yes	Yes	Yes	No	NA	8 – Good
Kadri et al. – 2004[40]	Yes	Yes	Yes	Yes	Yes	Yes	Yes	Yes	No	NA	8 – Good
Ueda et al. – 2006[44]	Yes	Yes	Yes	Yes	Yes	Yes	Yes	Yes	No	Yes	9 – Good
Chibbaro et al. – 2009[51]	Yes	Yes	Yes	Yes	Yes	Yes	Yes	Yes	No	NA	8 – Good
Ichimura et al. – 2009[53]	Yes	Yes	Yes	Yes	Yes	Yes	Yes	Yes	Yes	NA	9 – Good
Mathisien et al. – 2009[54]	Yes	Yes	Yes	Yes	Yes	Yes	Yes	Yes	No	NA	8 – Good
Watanabe et al. – 2009[55]	Yes	Yes	Yes	Yes	Yes	Yes	Yes	Yes	Yes	NA	9 – Good
Cavalcanti et al. – 2010[60]	Yes	Yes	Yes	Yes	Yes	Yes	Yes	Yes	Yes	NA	9 – Good
Takami et al. – 2010[61]	Yes	Yes	Yes	Yes	Yes	Yes	Yes	Yes	No	NA	8 – Good
Nonaka et al. – 2011[64]	Yes	Yes	Yes	Yes	Yes	Yes	Yes	Yes	No	NA	8 – Good
Wang et al. – 2011[65]	Yes	Yes	Yes	Yes	Yes	Yes	Yes	Yes	Yes	NA	8 – Good
Zhang et al. – 2011[66]	Yes	Yes	Yes	Yes	Yes	Yes	Yes	Yes	No	NA	8 – Good
Wan et al. – 2012[72]	Yes	Yes	Yes	Yes	Yes	Yes	Yes	Yes	No	NA	8 – Good
Zhang et al. – 2012[100]	Yes	Yes	Yes	Yes	Yes	Yes	Yes	Yes	No	NA	8 – Good
Chowdhury et al. – 2013[75]	Yes	Yes	Yes	Yes	Yes	Yes	Yes	Yes	Yes	NA	9 – Good
Xiao et al. – 2013[76]	Yes	Yes	Yes	Yes	Yes	Yes	Yes	Yes	Yes	NA	9 – Good
Nowak et al. – 2014[77]	Yes	Yes	Yes	Yes	Yes	Yes	Yes	Yes	No	NA	8 – Good

Suri et al. – 2014[78]	Yes	Yes	Yes	Yes	Yes	Yes	Yes	Yes	No	NA	8 – Good
Yu et al. – 2014[79]	Yes	Yes	Yes	Yes	Yes	Yes	Yes	Yes	No	NA	8 – Good
Hayashi et al. – 2015[81]	Yes	Yes	Yes	Yes	Yes	Yes	Yes	Yes	Yes	NA	7 – Good
Jiang et al. – 2015[82]	Yes	Yes	Yes	Yes	Yes	Yes	Yes	Yes	Yes	Yes	8 – Good
Goel et al. – 2018[92]	Yes	Yes	Yes	Yes	Yes	Yes	Yes	Yes	No	NA	8 – Good
Matsuda et al. – 2018[94]	Yes	Yes	Yes	Yes	Yes	Yes	Yes	Yes	No	NA	8 – Good
Aftahy et al. – 2021[99]	Yes	Yes	Yes	Yes	Yes	Yes	Yes	Yes	No	NA	8 – Good

References correspond to Supplementary File S1.
